# Comparative Metabolic Pathways Analysis and Subtractive Genomics Profiling to Prioritize Potential Drug Targets Against *Streptococcus pneumoniae*

**DOI:** 10.3389/fmicb.2021.796363

**Published:** 2022-02-10

**Authors:** Kanwal Khan, Khurshid Jalal, Ajmal Khan, Ahmed Al-Harrasi, Reaz Uddin

**Affiliations:** ^1^Dr. Panjwani Center for Molecular Medicine and Drug Research, International Center for Chemical and Biological Sciences, University of Karachi, Karachi, Pakistan; ^2^HEJ Research Institute of Chemistry, International Center for Chemical and Biological Sciences, University of Karachi, Karachi, Pakistan; ^3^Natural and Medical Sciences Research Center, University of Nizwa, Nizwa, Oman

**Keywords:** *Streptococcus pneumoniae*, subtractive genomics, serotype 14, metabolic pathways, 4-oxalocrotonate tautomerase and sensor histidine kinase

## Abstract

*Streptococcus pneumoniae* is a notorious pathogen that affects ∼450 million people worldwide and causes up to four million deaths per annum. Despite availability of antibiotics (i.e., penicillin, doxycycline, or clarithromycin) and conjugate vaccines (e.g., PCVs), it is still challenging to treat because of its drug resistance ability. The rise of antibiotic resistance in *S. pneumoniae* is a major source of concern across the world. Computational subtractive genomics is one of the most applied techniques in which the whole proteome of the bacterial pathogen is gradually reduced to a limited number of potential therapeutic targets. Whole-genome sequencing has greatly reduced the time required and provides more opportunities for drug target identification. The goal of this work is to evaluate and analyze metabolic pathways in serotype 14 of *S. pneumonia* to identify potential drug targets. In the present study, 47 potent drug targets were identified against *S. pneumonia* by employing the computational subtractive genomics approach. Among these, two proteins are prioritized (i.e., 4-oxalocrotonate tautomerase and Sensor histidine kinase uniquely present in *S. pneumonia*) as novel drug targets and selected for further structure-based studies. The identified proteins may provide a platform for the discovery of a lead drug candidate that may be capable of inhibiting these proteins and, therefore, could be helpful in minimizing the associated risk related to the drug-resistant *S. pneumoniae*. Finally, these enzymatic proteins could be of prime interest against *S. pneumoniae* to design rational targeted therapy.

## Graphical Abstract

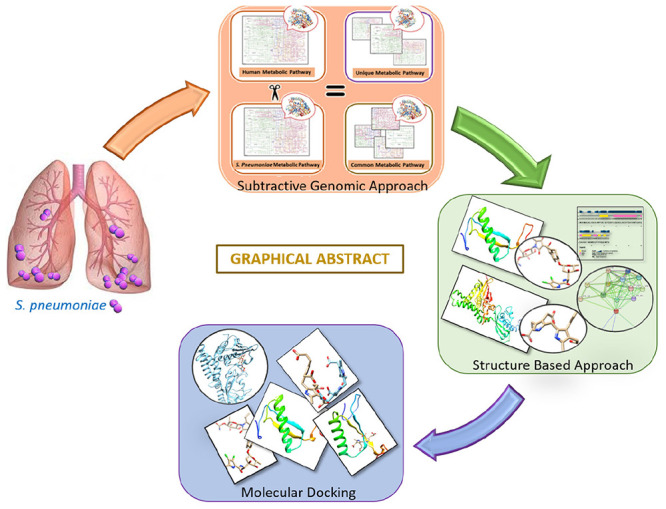

## Introduction

*Streptococcus pneumoniae* is a gram-positive, spherical, alpha-beta hemolytic, and facultatively anaerobic bacterium. It is classified primarily as one of the major pathogenic species resulting in high mortality and morbidity rate. It is responsible for causing meningitis, otitis media, septicemia, bacteremia, and community-acquired pneumonia, otherwise called Invasive Pneumococcal Disease (IPD) (Lo et al., 2019). Nearly ∼600,000 children, ∼200,000 elders, and immuno-compromised individuals die due to pneumococcal diseases caused by *streptococci* annually (Watt et al., 2009). Surprisingly, in 2019, >600,000 deaths were reported due to pneumonia as well as >385,000 deaths during the COVID-19 pandemic (Elflein, 2021). Antibiotics are used as the most common anti-infection therapy (e.g., beta-lactam antibiotics) for the treatment of pneumonia. Unfortunately, the increasing resistance against common antibiotics as well as the emergence of Multiple Drug Resistant strains (MDRs) worldwide makes the management and treatment of pneumococcal infections highly difficult (Hakenbeck et al., 2012). Surveillance of serotypes and prevalence of drug-resistant strains in the general population is critical to create appropriate prevention and treatment protocol for *S. pneumoniae* (Ediriweera et al., 2016).

Evidently, the serotype 14 is most frequently responsible for Invasive Pneumococcal Disease (IPD) (Geno et al., 2015) among the 101 defined *S. pneumoniae* serotypes. The conjugated pneumococcal vaccines are developed against *S. pneumoniae* infections based on the polysaccharide capsular serotypes (Chiba et al., 2014). Specifically, Polysaccharide Pneumococcal Vaccine (PPV) 23-valent was developed for serotype 14 to cure IPD. Unfortunately, PPSV23 led to poor immunogenicity for pneumococci (Cilloniz et al., 2016). Currently, there is Pneumococcal Polysaccharide Vaccine (PPSV23), 10-valent Pneumococcal Conjugate Vaccine (PCV10), 7-valent Pneumococcal Conjugate Vaccine (PCV7), and 13-valent Pneumococcal Conjugate Vaccine (PCV13) in use. Even after the distribution of multi-valent Pneumococcal Conjugate Vaccine (PCV7), the percentage of serotype 14 infection has increased over time due to the increase in drug resistance (Bouskraoui et al., 2011).

Experimentally, molecular serotyping such as MultiLocus Sequence Typing (MLST) and Pulse-Field Gel Electrophoresis (PFGE) are the gold standard methods used to study the outbreaks and identification of pneumococcal isolates (Enright and Spratt, 1998), but with high associated cost. Thus, accurate determination of the serotypes remained a challenge (Hu et al., 2014). It is therefore imperative that s novel therapeutic drug target is identified against *S. pneumoniae*. The discovery of a new drug target may lead to better therapeutics (Lodha et al., 2013). Fortunately, the arrival of the post-genomic era and whole-genome sequencing of the pathogens opened up new avenues, such as comparative subtractive genomics, to design new drugs and vaccine candidates. Computational approaches make it possible to identify potential drug targets against such pathogens (Fair and Tor, 2014).

Certain gaps from previous studies against *S. pneumoniae*, such as metabolic pathways coverage, consideration of hub nodes, and conserved drug targets of bacterial pneumonia (Henriques et al., 2000), are covered in this study. The current study includes comparative and subtractive genomics analysis, subcellular localization, Protein-Protein Interaction (PPI) network analysis, essentiality and druggability of the target proteins, and metabolic pathway analysis. Furthermore, the study proposed that antibacterial lead compounds could be developed against the shortlisted potential drug targets.

## Materials and Methods

**FIGURE 1:**
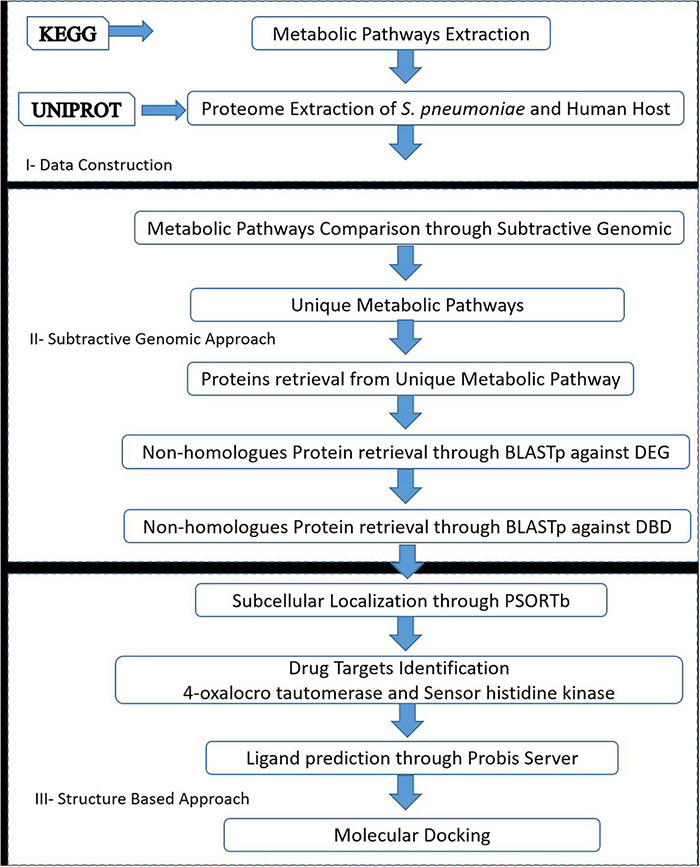
Flow chart: A general sketch of the current study integrated with the use of various computational approaches and tools to identify potential drug targets candidates.

The drug target prioritization and identification against *S. pneumoniae* was performed by employing a subtractive genomics approach along with the analysis of the metabolic pathways. Various databases and tools, as illustrated in the flow chart (Figure 1), were used for the determination of therapeutic targets against *S. pneumoniae.*

### Data Collection of Proteome and Metabolic Pathways

The Kyoto Encyclopedia of Genes and Genomes (KEGG) database (Kanehisa and Goto, 2000) was used for the metabolic pathways retrieval of the pathogen (*S. pneumoniae*, i.e., spw01100) and host (*Homo sapiens*, i.e., hsa01100). The human and *S. pneumoniae* proteomes were retrieved from the Universal Protein Resource (UniProt) database (Apweiler et al., 2021) with accession numbers UP000005640 and UP000001682 (strain CGSP14), respectively. The Database of Essential Genes (DEG database) (Luo et al., 2021) was used to investigate the essentiality of the drug targets and the DrugBank database (Wishart et al., 2018) was used to assess the druggability of the shortlisted targets.

### Subtractive Genomics Approach

In the current study, a “Subtractive Genomic Approach,” which is one of the most commonly applied methodologies, was applied. Subtractive Genomics is a powerful approach that applies the sequence subtraction between the host, the pathogen proteomes, and metabolic pathways. It provides necessary information on a set of proteins essential for the microorganism that do not exist in the respective host. Subtractive Genomics plays a role of great importance in potential drug target identification that is unique and essential to the pathogen survival without altering the host’s (human) systematic metabolic pathways (Fenoll et al., 2009).

### Host and Pathogen Metabolic Pathway Analysis

As mentioned above, the KEGG database (Kanehisa and Goto, 2000) was used for the genome-wide pathways analysis of *S. pneumoniae* and human. A standalone comparison was performed between the host and pathogen to identify unique and commonly found metabolic pathways. Unique pathways are classified as those pathways that are present only in the pathogen, while common pathways are those pathways that are present in both organisms (i.e., pathogen and host) (Linares et al., 2010). In the present study, the common metabolic pathways proteins were discarded while the protein sequences of unique metabolic pathways were retrieved from the UniProt for further downstream study.

### Non-homologous Protein Identification

Accordingly, the protein sequences retrieved from the unique metabolic pathways were subjected to BLASTp (with *E*-value 10-3) against *Homo sapiens*. The BLASTp resulted in “Hits” (Homologous sequences between host and pathogen) and “No-Hits” (Non-homologous sequences). For further analysis, the non-homologous sequences that showed no similarity with the human host were selected.

### Essential Non-homologous Genes Identification

Proteins that play a major role in cellular metabolism are said to be essential for any organism’s survival (Deng et al., 2011). Thus, to shortlist proteins essential to pathogen’s survival, BLASTp of non-homolog *S. pneumoniae* proteins was performed against DEG with cut-off parameters of *E* value 10^–5^. Consequently, the obtained Hits protein sequences (homologous proteins) were used for further analysis while “No-Hits” (non-homologous proteins) protein sequences were discarded.

### Druggability of Essential Protein

Furthermore, the essential non-homologous proteins were assessed through BLASTp with an *E* value 10^–3^ against the DrugBank database to determine the drug–target-like ability of shortlisted proteins to finally identify novel drug targets against *S. pneumoniae*. Similarly, the identified Hits protein sequences were retrieved and analyzed for the prioritization of a potent drug target while obtained “No-Hits” proteins sequences were discarded.

### Sub-Cellular Location Prediction

The subcellular location of final shortlisted drug-like protein targets was determined by using PSORTb v.3.0. tool (Yu et al., 2010). The PSORTb predicts results based on the sub-cellular localization, i.e., cytoplasmic membrane, cell wall, cytoplasmic, extracellular, and unknown.

### Protein Structure Prediction, Validation, and Conservancy Analysis

The structures from the proteins shortlisted from the above approach were searched for in PDB. The suitable template for the protein structure modeling was found through BLASTp against the PDB. It resulted in the shortlisting of various proteins with different query coverage and percent identity that can be further filtered to select a suitable template for protein structure modeling. Eventually, the 3D structures of shortlisted drug targets were then modeled by using the Homology modeler tool. Based on DOPE score, the best optimized structure from five modeled structures was selected. In order to perform the docking experiment against shortlisted proteins, PSIPRED (Buchan and Jones, 2019), ProSA-web (Wiederstein and Sippl, 2007), and PROCHECK (Laskowski et al., 2006) were applied for the model structure evaluation based on secondary structure analysis, error in 3D model identification, and tertiary structure stereochemistry analysis, respectively.

The shortlisted proteins were further analyzed to investigate the conservancy of these proteins in the *S. pneumoniae* strains. Therefore, BLASTp of shortlisted proteins was performed and resultant matched sequences were retrieved. These sequences were further aligned through Clustal Omega tool to study conservation among these proteins from other serotypes of *S. pneumoniae*.

### Active Site Prediction

As the structure was modeled, there is a need to find the active site where a ligand could bind to alter its function. The DogSite Scorer^1^ was used to find the possible binding sites of the modeled protein. The DogSite Scorer identifies active site pockets based on the physico-chemical properties of the protein residues. These active sites can be selected to dock ligand against respective proteins.

### Network Topology Studies

Furthermore, to identify whether these proteins can be a hub protein and validate their functional interactions, the PPIs network of pathogen proteins as potential drug targets were generated through the STRING database (Szklarczyk et al., 2021). The STRING is a database of experimentally known and predicted PPIs, including direct (physical) and indirect (functional) network. Node degrees and clustering coefficient are used to classify these PPIs as hub proteins.

### Ligand Prediction

As the protein structure was not available, the ligand information was also missing. The ProBis server (Konc et al., 2015) was used to find the probable ligands and also used for the assessment of the fundamental interaction between the ligand and drug targets. The ProBis predicts the best possible ligand for proteins through molecular simulation based on a theoretical approach. On the basis of the proposed ligand, one may design new drugs in further drug discovery stages.

### Molecular Docking Studies

In molecular docking, the most effective ligand shows the minimal score of docking for its target proteins. The proteins with the modeled structure were used as target proteins while identified ligands were docked. The ligand was docked following the standard docking parameters of 250 times Lamarckian GA settings resulting in 27,000 generations (Tanchuk et al., 2016). The MOE software was used to investigate the docked ligand-protein interactions, and to visualize the hydrogen bond and hydrophobic interactions of the ligand with docked protein within the range of 5 Å.

## Results

### Identification of Metabolic Pathways in *Streptococcus pneumoniae*

Currently, the KEGG database has 115 metabolic pathways for *S. pneumoniae* serotype 14 and 375 for human, as shown in Table 1. Unique and common metabolic pathways among pathogen and host (human) were identified through standalone BLASTp. Consequently, 25 unique pathways are identified for the pathogen that are crucial for the persistence of *S. pneumoniae* (Table 2) with 90 pathways commonly present in both organisms (Table 3).

**TABLE 1:** Steps involved in the current study: Subtractive filtering of proteins and metabolic pathways against *S. pneumoniae*.

S. no	Steps involved in the current study	*Streptococcus pneumoniae*
1	Complete pathways of the pathogen from KEGG (spw)	115
2	Complete pathways of the human from KEGG (hsa)	375
3	Common metabolic pathways	90
4	Unique metabolic pathways	25
5	Number of proteins presents in unique metabolic pathways	318
6	Removal of redundant KEGG IDs	207
7	BLASTp of unique metabolic proteins against human host proteome (*E* value 10^–3^)	150
8	BLASTp of unique metabolic proteins against DEG (*E* value 10^–5^)	105
9	BLASTp of unique metabolic proteins against DBD (*E* value 10^–3^)	47

**TABLE 2:** Unique metabolic pathways: List of all unique metabolic pathways and uniquely present proteins in these metabolic pathways present in *S. pneumoniae*.

S. no	Metabolic pathways	Pathway Ids	Proteins in the pathway
1	Aminobenzoate degradation	spw00627	2
2	Antimicrobial resistance genes	spw01504	17
3	Bacterial secretion system	spw03070	13
4	Benzoate degradation	spw00362	2
5	C5-Branched dibasic acid metabolism	spw00660	5
6	Carbapenem biosynthesis	spw00332	2
7	Cationic antimicrobial peptide (CAMP) resistance	spw01503	6
8	Chloroalkane and chloroalkene degradation	spw00625	2
9	Cyanoamino acid metabolism	spw00460	4
10	D-Alanine metabolism	spw00473	4
11	Lysine biosynthesis	spw00300	12
12	Methane metabolism	spw00680	9
13	Monobactam biosynthesis	spw00261	4
14	Naphthalene degradation	spw00626	2
15	Peptidoglycan biosynthesis	spw00550	25
16	Peptidoglycan biosynthesis and degradation protein	spw01011	24
17	Phosphotransferase system	spw02060	45
18	Photosynthesis proteins	spw00194	8
19	Quorum sensing	spw02024	52
20	Streptomycin biosynthesis	spw00521	3
21	Two-component system	spw02022	37
22	Two-component system	spw02020	16
23	Vancomycin resistance	spw01502	6
24	Xylene degradation	spw00622	2
25	beta-Lactam resistance	spw01501	16

**TABLE 3:** Common metabolic pathways: List of all common metabolic pathways, commonly shared by both organisms (*S. pneumoniae* and Human).

S. no	Metabolic pathways	Pathway ids	Protein in pathways
1	ABC transporters	pw02010	97
2	Alanine, aspartate, and glutamate metabolism	spw00250	17
3	Amino acid related enzymes	spw01007	27
4	Amino sugar and nucleotide sugar metabolism	spw00520	35
5	Aminoacyl-tRNA biosynthesis	spw00970	84
6	Arachidonic acid metabolism	spw00590	1
7	Arginine and proline metabolism	spw00330	7
8	Arginine biosynthesis	spw00220	7
9	Ascorbate and aldarate metabolism	spw00053	11
10	Bacterial toxins	spw02042	4
11	Base excision repair	spw03410	9
12	Biotin metabolism	spw00780	5
13	Butanoate metabolism	spw00650	7
14	CD molecules	spw04090	1
15	Chaperones and folding catalysts	spw03110	18
16	Chloroalkane and chloroalkene degradation	spw00625	2
17	Chromosome and associated proteins	spw03036	29
18	Citrate cycle (TCA cycle)	spw00020	4
19	Cysteine and methionine metabolism	spw00270	21
20	Cytoskeleton proteins	spw04812	3
21	D-Glutamine and D-glutamate metabolism	spw00471	3
22	DNA repair and recombination proteins	spw03400	67
23	DNA replication	spw03030	15
24	DNA replication proteins	spw03032	25
25	Enzymes	spw01000	1
26	Exosome	spw04147	26
27	Fatty acid biosynthesis	spw00061	15
28	Folate biosynthesis	spw00790	9
29	Fructose and mannose metabolism	spw00051	20
30	Galactose metabolism	spw00052	27
31	Glutathione metabolism	spw00480	8
32	Glycerolipid metabolism	spw00052	10
33	Glycerophospholipid metabolism	spw00564	8
34	Glycine, serine, and threonine metabolism	spw00260	16
35	Glycolysis/Gluconeogenesis	spw00010	26
36	Glycosyltransferases	spw01003	6
37	Glyoxylate and dicarboxylate metabolism	spw00630	7
38	Homologous recombination	spw03440	21
39	Inositol phosphate metabolism	spw00562	4
40	Ion channels	spw04040	1
41	Lipid biosynthesis proteins	spw01004	12
42	Lysine degradation	spw00310	2
43	Membrane trafficking	spw04131	5
44	Messenger RNA biogenesis	spw03019	14
45	Mismatch repair	spw03430	18
46	Mitochondrial biogenesis	spw03029	27
47	Nicotinate and nicotinamide metabolism	spw00760	8
48	Nitrogen metabolism	spw00910	4
49	Non-coding RNAs	spw03100	74
50	Nucleotide excision repair	spw03420	8
51	One carbon pool by folate	spw00670	10
52	Other glycan degradation	spw00511	10
53	Oxidative phosphorylation	spw00190	18
54	Pantothenate and CoA biosynthesis	spw00770	11
55	Pentose and glucuronate interconversions	spw00040	9
56	Pentose phosphate pathway	spw00030	19
57	Peptidases and inhibitors	spw01002	38
58	Phenylalanine, tyrosine, and tryptophan biosynthesis	spw00400	18
59	Porphyrin and chlorophyll metabolism	spw00860	1
60	Prenyltransferases	spw01006	3
61	Prokaryotic defense system	spw02048	25
62	Propanoate metabolism	spw00640	12
63	Protein export	spw03060	14
64	Protein kinases	spw01001	10
65	Protein phosphatases and associated proteins	spw01009	3
66	Purine metabolism	spw00230	36
67	Pyrimidine metabolism	spw00240	30
68	Pyruvate metabolism	spw00620	22
69	RNA degradation	spw03018	10
70	RNA polymerase	spw03020	6
71	Riboflavin metabolism	spw00740	6
72	Ribosome	spw03011	63
73	Ribosome biogenesis	spw03009	52
74	Secretion system	spw02044	13
75	Selenocompound metabolism	spw00450	7
76	Sphingolipid metabolism	spw00600	6
77	Starch and sucrose metabolism	spw00500	40
78	Sulfur metabolism	spw00920	5
79	Sulfur relay system	spw04122	5
80	Taurine and hypotaurine metabolism	spw00430	4
81	Terpenoid backbone biosynthesis	spw00900	8
82	Thiamine metabolism	spw00730	13
83	Transcription factors	spw03000	41
84	Transcription machinery	spw03021	11
85	Transfer RNA biogenesis	spw03016	52
86	Translation factors	spw03012	14
87	Transporters	spw02000	264
88	Valine, leucine, and isoleucine biosynthesis	spw00290	11
89	Valine, leucine, and isoleucine degradation	spw00280	3
90	Vitamin B6 metabolism	spw00750	4

### Metabolic Pathway Analysis

Furthermore, BLASTp of proteins that are uniquely present in the shortlisted unique metabolic pathways was performed against the human proteome to encounter further cross-reactivity of drug-like compounds with the host protein. Metabolic pathway analysis through KEGG showed 318 proteins present in the unique metabolic pathways in which the shortlisted proteins are playing significant roles, as shown in Table 2.

### Non-homologous Proteins Identification

As described above, a total of 318 proteins were retrieved from the unique metabolic pathways. These 318 proteins were subjected to BLASTp to determine the non-homologous protein sequences against the host proteome. The BLASTp revealed 150 proteins classified as non-homologous proteins that are present only in the pathogen. These proteins are further analyzed in subsequent steps.

### Identification of Essential Proteins

The essentiality of all non-homologous proteins was determined by using BLASTp from the DEG database with the *E* value of 10^–5^. About 105 non-homolog proteins were classified as essential proteins required for the survival of *S. pneumoniae* and could be proposed as the potential drug targets (Supplementary Table 1). These non-homologous essential proteins can be safely recommended as possible therapeutic targets for pathogens. Theoretically, bacteria may survive but expected to be less virulent if such proteins are targeted, or numerous important processes could be inhibited, resulting in pathogenicity being eradicated. The DEG database is being updated periodically, however, it is limited to the studies of *S. pneumoniae* survival in rich growth medium only. Despite the DEG’s limitation it is a well cited database and provided reliable results for known organisms. The essentiality of *S. pneumoniae* proteins can be further evaluated through experimental studies for the survival of pathogen in environmental or other biological conditions such as saliva (van Opijnen et al., 2009; Liu et al., 2017, 2021).

### Druggability of Therapeutic Targets

Eventually, the non-homologous essential 105 proteins were BLASTp-ed against the DrugBank database and any sequence similarities with the drug target proteins in the DrugBank were found. Only proteins with significant sequence similarity to FDA-approved therapeutic targets were chosen, while the remaining were eliminated from the database. As a result, only 47 proteins were identified as being essential, non-homologous, and drug target-like against *S. pneumoniae*. These 47 proteins showed significant similarities with the FDA approved drug targets found in DrugBank and, therefore, subsequently followed up in the next step. On the other hand, the excluded 58 proteins at this stage have not shown any significant similarity to the drug targets found in DrugBank. Although those 58 proteins were excluded they still may be studied as potential drug targets by the scientific community owing to their essentiality and non-homologous nature. The list of these 47 drug target-like proteins are provided in Supplementary Table 2.

### Subcellular Localization Prediction

**FIGURE 2:**
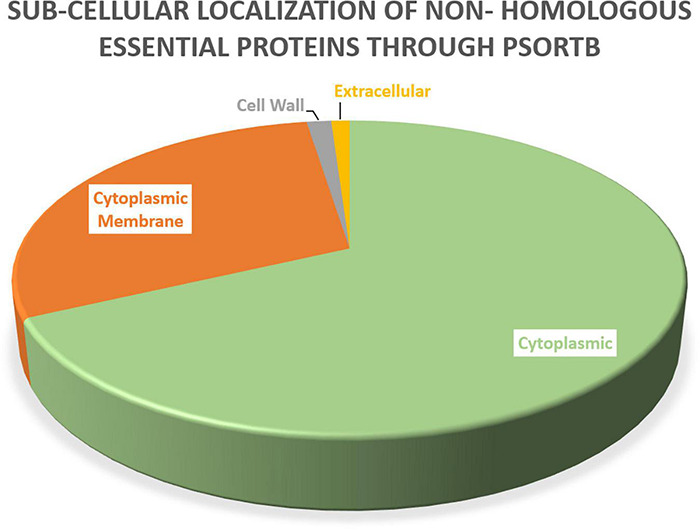
Subcellular localization: PSORTb results showing the subcellular distribution of essential proteins occur in *S. pneumoniae*.

Protein localization is important to understand throughout the drug development process because it influences the design of novel drugs and vaccines. Cell membrane proteins, for example, are frequently employed as vaccine targets, while cytoplasmic proteins are frequently used as therapeutic targets. Among these 47 essential proteins, 30 were found to be cytoplasmic proteins, 11 were cytoplasmic membrane proteins, three were cell wall proteins, and one was identified as an extracellular protein, as shown in Table 4. Figure 2 showed the location distribution of all essential proteins in *S. pneumoniae*. The step-wise filtering of the proteins during the current study is shown in Table 1.

**TABLE 4:** Subcellular localization: Distribution of essential non-homologous proteins in a different area of cell.

S. no	PSORTb results	No. of proteins
1	Cell wall	3
2	Cytoplasmic	30
3	Cytoplasmic Membrane	11
4	Extracellular	1
5	Unknown	2

### Novel Drug Targets Prediction and Significance of Selected Proteins

**FIGURE 3:**
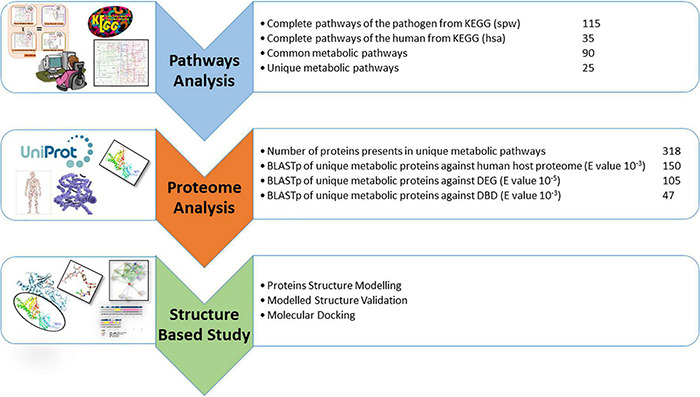
Current study summary for target protein identification: Stepwise analysis of subtractive genomic approach for drug targets identification in *S. pneumoniae*.

The literature is full of examples of cytoplasmic proteins as proven therapeutic targets because of easy reach by the drugs (Anis Ahamed et al., 2021). Additionally, ∼70% FDA approved drugs are reported as enzymatic proteins because of their significant role in multiple pathways. Finally, among 47 potential drug targets, two proteins were shortlisted as essential, non-homologous, druggable targets against *S. pneumoniae* i.e., 4-oxalocrotonate tautomerase (B2IPH4) and sensor histidine kinase CiaH (B2INS3). Based on their cytoplasmic subcellular localization, length > 100 amino acids, their enzymatic nature, and involvement in essential metabolic pathways, these identified proteins were subjected to further structure-based studies. Figure 3 showed the comprehensive outcome of the current study.

### 4-Oxalocrotonate Tautomerase

4-oxalocrotonate tautomerase enzyme (EC 5.3.2.-4-OT) belongs to a family of isomerases that readily convert hydroxymuconate to the αβ-unsaturated ketone (van Diemen et al., 2017). It is involved in benzonate degradation and xylene degradation pathways uniquely found in *S. pneumoniae*.


$$\text{Aromatic}\text{compound}\overset{4-\mathrm{OT}}{⟶}\alpha ,\beta -unsaturated\text{ketone}$$

Through this enzyme, bacteria utilize aromatic compounds and amino acids to essential hydrocarbons which are the sole source of carbon and energy that is further used in the citric acid cycle.

### Sensor Histidine Kinase CiaH

Sensor histidine kinase CiaH (EC:2.7.13.3) is an ATP binding signal transduction protein involved in the Two-component system of *S. pneumoniae* (Singh et al., 2011). These sensor histidine kinases sense the changes in the environment (in stress or presence of drug) surrounding the pathogen and provide such signals that alter the inside mechanism of bacterial cells, preparing it to utilize these changes. It has been reported that the changes in these sensor kinases showed resistance to many antibacterial drugs such as cefotaxime (Möglich, 2019). The protein helps in the catalysis of reactions such as,


$$\mathrm{ATP}+\mathrm{protein}\text{L-histidine}\overset{\text{CiaH}}{⟶}\mathrm{ADP}+\mathrm{protein}$$

N-phospho-L-histidine

Environmental variables influence the synthesis of virulence proteins, and two-component regulatory mechanisms are involved in detecting these influences. Tatsuno et al. (2014) reported the effected regulation of Streptococcus and significant growth reduction in knockout CiaH strains. Additionally, CiaH is highly conserved in *S. pneumoniae*, and deletion of the gene encoding its cognate response regulator (ciaR*pn*) made pneumococcal strains more susceptible to oxidative stress (Kaiser et al., 2020). Furthermore, it has been widely studied for its mutations resulting in the beta-lactamase resistance (Schweizer et al., 2017; Peters et al., 2021). It is used as a drug target to inhibit *ESKAPE* pathogens (Velikova et al., 2016), *Staphylococcus aureus* (Xie et al., 2020), and *Corynebacterium diphtheriae* (Khalid et al., 2018). However, CiaH protein has never been studied as drug target against *S. pneumoniae* and thus in the current study it is proposed as a potential drug target.

### Conservancy Analysis of Shortlisted Proteins

Furthermore, the conservancy analysis was performed for 4-oxalocrotonate tautomerase and sensor histidine kinase CiaH proteins. The BLASTp of 4-oxalocrotonate tautomerase resulted in the local alignment of XylH with P67530 *S. pneumoniae* (serotype 4), P67531 (strain ATCC BAA-255/R6), A5MAV1 (strain SP14-BS69), and J0V2K5 (from strain 2070335). The alignments of these identified proteins were further analyzed through multiple sequence alignment using Clustal Omega. The percent matric analysis showed that these proteins are 100–98% conserved among them (Supplementary Figure 1). Additionally, the BLASTp of sensor histidine kinase CiaH showed similarities with P0A4I6 (strain ATCC BAA-255/R6), P0A4I5 (serotype 4 strain ATCC BAA-334/TIGR4), A0A0H2ZQ10 (serotype 2 strain D39/NCTC 7466), J1DIP7 (strain 2070335), A5M9S1 (SP14-BS69), and A5M9S2 (strain SP14-BS69), respectively. These proteins showed conservancy of 100–93% when analyzed through MSA (Supplementary Figure 2).

### Comparative Structure Prediction and Evaluation

**FIGURE 4:**
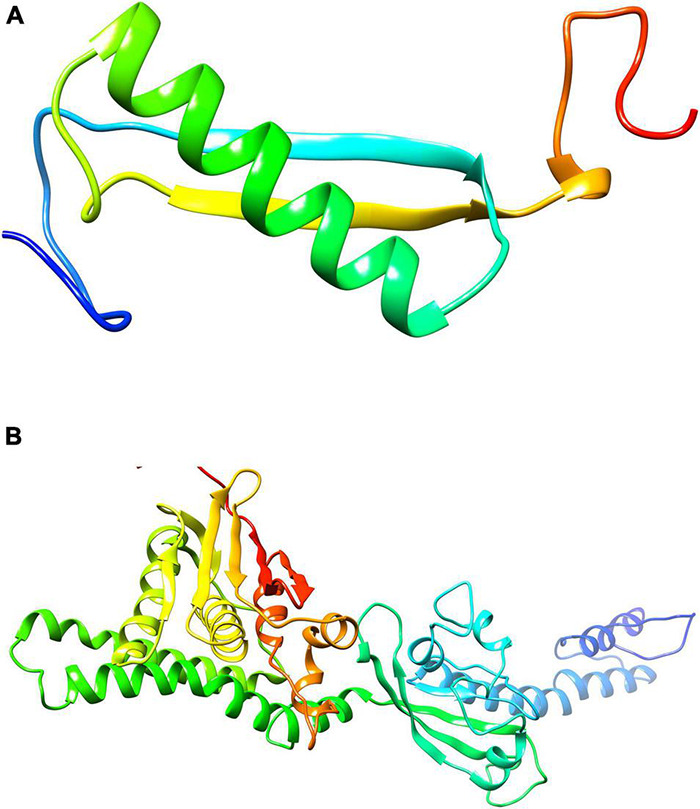
Modeled structure of proteins (drug targets): Structure modeled through Homology modeler for 4-oxalocrotonate tautomerase **(A)** and sensor histidine kinases **(B)** using the respective template.

The 3D structures of shortlisted proteins were not yet available in the PDB. Therefore, its homology modeling was performed by taking the FASTA sequence of the protein from the UniProt database with the accession numbers B2IPH4 and B2INS3 as mentioned in the database. Structural and functional studies of 4-oxalocrotonate tautomerase and sensor histidine kinase CiaH, were further evaluated by performing BLASTp against the PDB database to find a possible template. For 4-oxalocrotonate tautomerase, the template PDB ID: 6FPS was identified with 41% sequence similarity (Figure 4A). Likewise, for sensor histidine kinase CiaH, template PDB ID: 4I5S with a 40% sequence similarity was selected (Figure 4B). Using these identified template proteins, the 3D structure of 4-oxalocrotonate tautomerase and sensor histidine kinase CiaH was modeled. Structures with high DOPE score (i.e., modeled structure 4) were further evaluated for ligand screening.

### Validation of the Modeled Structure

Different tools were used to verify the modeled protein structure. In the following, the structure verification procedure is discussed.

#### Confirmation of Proteins Through PSIPRED

The PSIPRED resulted in the prediction of a higher number of alpha helices than beta sheets formation as shown in Supplementary Figure 3 for both XylH and CiaH. The PSIPRED results verified the protein on the basis of their sequence for the alpha helices and beta sheets formation as modeled through modeler tool.

#### PROCHECK Validation of Proteins

The PROCHECK was used to generate a Ramachandran plot for the modeled protein structures. For XylH, the Ramachandran plot showed about 93.8% residues in the favorable region and four residues in additionally allowed regions (6.2%). While in the case of CiaH, validation showed about 84.4% residues in the favorable region, with seven residues in the disallowed region and 51 and 7 residues in the additionally allowed and generously allowed regions responsible for about 12.2 and 1.7%, respectively, as shown in Supplementary Figure 4.

#### Structure Validation Through ProSA

Additionally, the cross validation of modeled structure through ProSA tool predicted a *Z*-score value of –2.66 for XylH and –4.62 for CiaH, indicating the model falls within the range of NMR/X-ray crystallography derived structures (Supplementary Figure 5).

### Network Topology for Protein-Protein Interactions

Many functional and physical interactions between various types of proteins develop, and these interactions are critical for many biological processes involving cellular machinery. Filtering and analyzing functional genomic data for annotating functional, structural, and evolutionary information of proteins may be performed using this information. Investigating the predicted PPIs might open up new avenues for experimental research and computer-assisted drug discovery in the future (Schwartz et al., 2009). The PPI and functional annotation of selected proteins (XylH and CiaH) were determined using the STRING server. The STRING results showed different nodes and edges of each protein and showed that the prioritized target proteins may act as hub protein inter-acting with more than ten proteins. Therefore, targeting such proteins may affect the activity of all interactor proteins.

**FIGURE 5:**
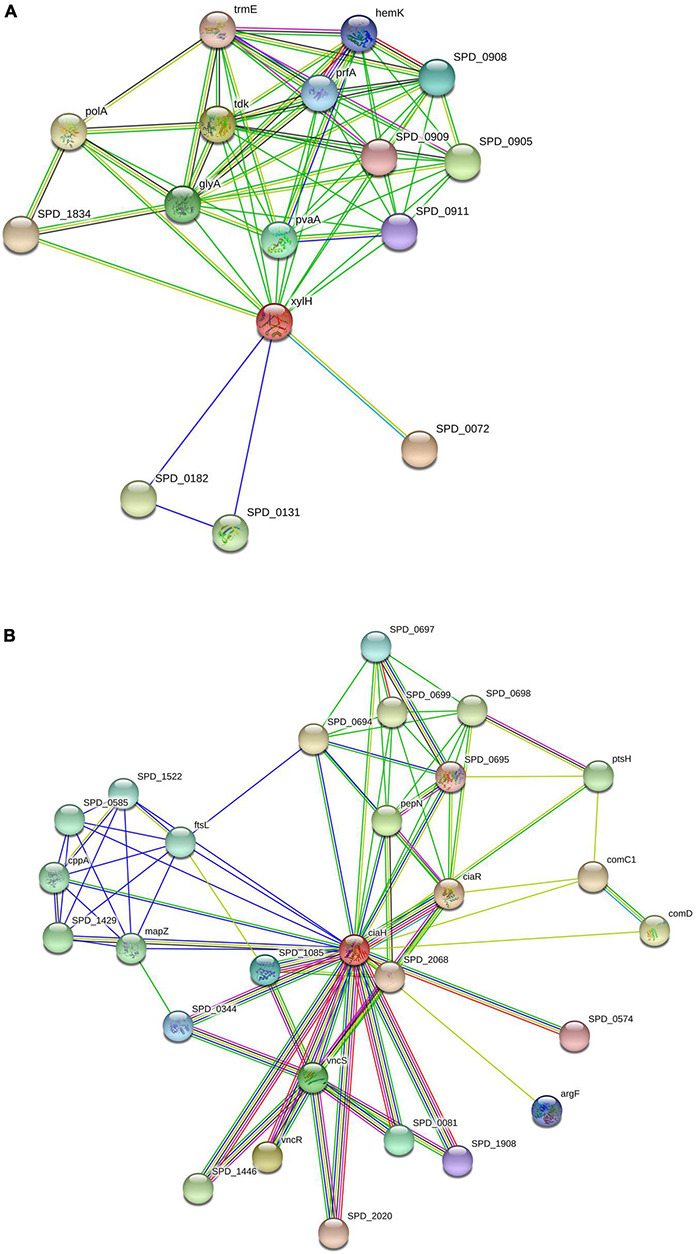
Protein-protein interactions: Schematic PPI network generated through the STRING database for XylH **(A)** and CiaH **(B)**.

The interaction of XylH protein with other proteins in *S. pneumoniae* was identified using the STRING database, which was submitted with the protein sequence of XylH. It resulted in 16 PPI networks for 4-oxalocrotonate tautomerase (Figure 5A) represented as (XylH in red node) with neighbor proteins as SPD_0131 (Conserved hypothetical protein), SPD_0182 (Conserved hypothetical protein), SPD_0072 (Catechol 2,3-dioxygenase), SPD_1834 (Acetaldehyde dehydrogenase), TrmE (Trna modification gtpase trme), glyA (Glycine hydroxymethyltransferase), PolA (In addition to polymerase activity), prfA (Peptide chain release factor 1), tdk (Thymidine kinase), SPD_0905 (Acetyltransferase), SPD_0908 (L-threonylcarbamoyladenylate synthase), SPD_0911 (Uncharacterized protein), PvaA (Pneumococcal vaccine antigen A), and HemK (Release factor glutamine methyltransferase). The XylH protein has a total number of edges of 62, expected number of edges of 23, number of nodes of 16, and an average nodes degree of 7.75, according to the results. The enrichment *p*-value for Protein-Protein Interactions is 7.79e-12, with an average local clustering coefficient of 0.908. These proteins are involved in a variety of critical functions. As a result, targeting the XylH protein may result in the loss of function of the other associated proteins. As a result, we can suggest this protein as a therapeutic target.

Similarly, the CiaH protein sequence was uploaded to the STRING database. The STRING resulted in 28 PPI networks for sensor histidine kinase CiaH, verifying that the selected proteins are hub proteins (Figure 5B). The CiaH was represented by a red node having interactions with CiaR (DNA-binding response regulator ciaR), SPD_2068 (DNA-binding response regulator), SPD_0574 (DNA-binding response regulator), ArgF (Ornithine carbamoyltransferase), SPD_1908 (Response regulator), SPD_0081 (Response regulator), VncS (Histidine kinase), VncR (DNA-binding response regulator VncR), SPD_1446 (DNA-binding response regulator), SPD_2020 (DNA-binding response regulator protein), SPD_0344 (DNA-binding response regulator), SPD _1085 (response regulator saer), SPD_0574 (DNA-binding response regulator), ComD (sensor histidine kinase comd), ComC1 (Competence stimulating peptide precursor 1), PtsH (phosphocsrrier protein), SPD_0698 (Uncharacterized protein), SPD_0695 (Oxidoreductase), SPD_0699 (Uncharacterized protein), SPD_0694 (Uncharacterized protein), SPD_0697 (Acetyltransferase), PepN (Aminopeptidase), mapZ (Conserved hypothetical protein), SPD_1429 (Uncharacterized protein), CppA (C_3_-degrading proteinase), SPD_0585 (Uncharacterized protein), SPD_1522 (Replication initiation and membrane attachment protein), and FtsL (Cell division protein ftsl). It has a total of 28 number of nodes, 90 edges, 6.43 average node degree, average clustering coefficient as 0.824, expected number of edges as 38, and PPI enrichment *p*-value of 6.97e-13.

### Active Site Prediction

Accordingly, as the protein structures were modeled, there is a need to find an interactive interface for the binding of the ligand. For that purpose, the DogSite Scorer tool was used to identify such binding sites. It predicted only one binding pocket for XylH protein with a drug score of 0.82 as shown in Supplementary Figure 6A. Table 5 showed the residues present in the selected binding pocket. As for CiaH, it predicted 16 binding pockets. Among these pockets, the binding site with a high drug score 0.81 was selected, as shown in Supplementary Figure 6B. Table 5 showed the residues present in the selected binding pocket.

**TABLE 5:** Active site: Residues present in the Active site of XylH and CiaH protein.

XylH protein active site residues				CiaH protein active site residues		
S. no	Chain	Position	Residue	Chain	Position	Residue
1	A	1	Met	A	211	Leu
2	A	2	Val	A	212	Glu
3	A	3	Lys	A	218	Gln
4	A	4	Trp	A	219	Ser
5	A	5	Lys	A	223	Asn
6	A	6	Lys	A	226	His
7	A	7	Ser	A	227	Glu
8	A	8	Lys	A	228	Leu
9	A	9	Leu	A	229	Arg
10	A	10	Val	A	230	Thr
11	A	11	Glu	A	231	Pro
12	A	12	Glu	A	233	Ala
13	A	13	Ala	A	234	Val
14	A	14	Ile	A	235	Leu
15	A	15	Met	A	236	Gln
16	A	16	Pro	A	237	Asn
17	A	17	Phe	A	238	Arg
18	A	28	Leu	A	256	Ser
19	A	31	Lys	A	259	Ser
20	A	32	Lys	A	260	Ser
21	A	35	Ala	A	262	Glu
22	A	36	Lys	A	263	Glu
23	A	39	Thr	A	266	Asn
24	A	48	Ala	A	267	Met
25	A	49	Pro	A	269	Phe
26	A	50	Gln	A	270	Leu
27	A	51	Ser	A	273	Ser
28	A	52	Ala	A	274	Leu
29	A	53	Val	A	280	Arg
30	A	54	His	A	281	Asp
31	A	56	Val	A	282	Asp
32				A	284	Ile
33				A	289	Ala
34				A	290	Glu
35				A	294	Ser
36				A	295	Phe
37				A	298	Thr
38				A	301	Thr
39				A	332	Lys
40				A	351	Glu
41				A	385	Arg
42				A	402	Leu
43				A	407	Ala
44				A	410	Ile
45				A	413	Ala

### Protein-Ligand Interactions Study Through Docking

The protein-ligand interactions were analyzed through AutoDock Vina.

#### Ligand Identification

The identification of protein binding site and their corresponding ligands have an important role in drug target identification and drug research. The protein binding sites are structurally and functionally important regions on the protein surface on which different type of drugs interact to perform a desired action (Konc and Janežič, 2017). The ProBis server was used for the ligand identification. For 4-oxalocrotonate tautomerase, DPM commonly named as DIPYRROMETHANE COFACTOR with IUPAC name: 3-[5-[[3-(2-carboxyethyl)-4-(carboxymethyl)-5-methyl-1H-pyrrol-2-yl]methyl]-4-(carboxymethyl)-1H-pyrrol-3-yl] propanoic acid was used. The ligand was identified from a template protein PDB ID: 3EQ1 (from Human) and for sensor histidine kinase CiaH, XAM commonly named as Amycolamicin antibiotic, IUPAC name (1R,4aS,5S,6S,8aR)-5-{[(5S)-1-(3-O-acetyl-4-O-carbamoyl-6-deoxy-2-O-methyl-alpha -L-talopyranosyl)-4-hydroxy-2-oxo-5-(propan-2-yl)-2,5-dihydro-1H-pyrrol-3-yl]carbonyl}-6-methyl-4-methylidene-1,2, 3,4,4a,5,6,8a-octahydronaphthalen-1-yl-2,6-dideoxy-3-C-[(1S)- 1-{[(3,4-dichloro-5-methyl-1H-pyrrol-2-yl)carbonyl]amino} ethyl]-beta-D-ribo-hexopyranosideligand was identified from a template PDB ID: 4URL (from *Escherichia coli* BL21) (Supplementary Figure 7).

#### Molecular Docking With AutoDock

**FIGURE 6:**
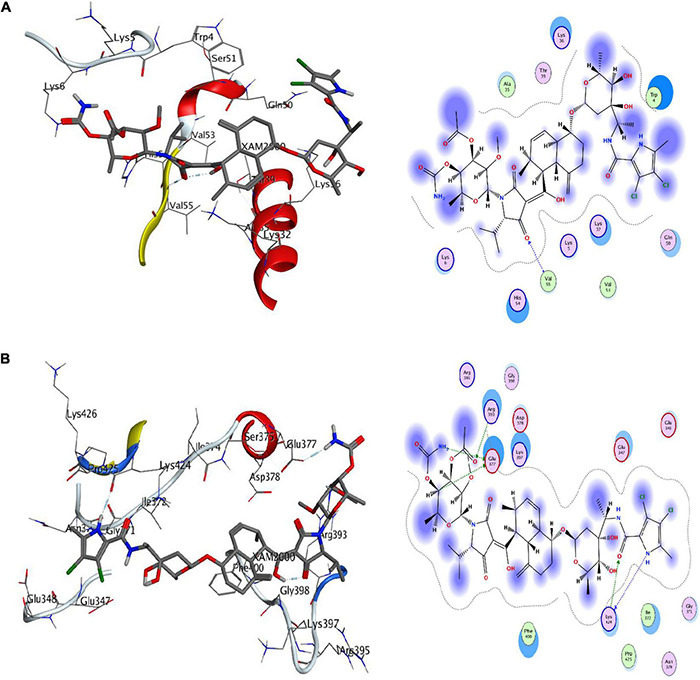
Docking of ligands with their respective drug targets. **(A)** 3D and 2D interaction of DPM with Ciah protein, and **(B)** XAM ligand for XylH protein.

Molecular docking analysis was performed with the identified ligand from ProBis server and modeled structure of CiaH and XylH protein through the AutoDock 4.2 tool. The XAM compound resulted in the binding score of –5.09 kcal/mol while DPM showed more potency toward XylH i.e., –7.59 kcal/mol. Furthermore, the post-docking analysis was performed to study the interaction of protein compounds complex. The analysis showed that XAM mediates five hydrogen bonds with Glu377, Lys424, and Arg393 whereas DPM mediates one hydrogen bond through its side chain oxygen with Val55 residue, as shown in Figure 6. The detailed interaction analysis of XAM and DPM is highlighted in Table 6.

**TABLE 6:** Post-docking interactional analysis of XAM and DPM with XylH and CiaH protein.

Compounds	Ligand	Receptor	Interaction	Distance	E (kcal/mol)	Binding affinity (kcal/mol)
XAM	C4 1	OE2 GLU377	H-donor	3.21	–0.7	–7.59
	NAV 20	O LYS424	H-donor	2.98	–4.5	
	NCA 115	OE2 GLU377	H-donor	3.09	–5.0	
	OAQ 24	CB LYS424	H-acceptor	3.54	–0.7	
	OBY 120	NH2 ARG393	H-acceptor	3.45	–0.6	
DPM	OBI 88	N VAL55	H-acceptor	3.11	–2.6	–5.09

## Discussion

*Streptococcus pneumoniae* is the most common cause of pneumonia in children and the number of antimicrobial-resistant cases of *S. pneumoniae* has increased globally. The situation tends to the worst due to limited medication for acute and chronic illnesses (Yang et al., 2019). Recently, computational methods gained more attention for the development of numerous alternative approaches for treating the resistant pathogens (Boeckmann et al., 2003). The subtractive genomics has been widely applied on various pathogens for the prediction and identification of therapeutic targets. Despite the methodological advancements, the high throughput experimental results are yet not available for the majority of pathogens. As a result, the efforts to find essential drug targets now mostly rely on bioinformatics based predictions (Uddin and Rafi, 2017). *In silico* based subtractive genomic analysis has been widely applied for strain-specific drug target identifications, particularly for the resistant pathogens (Uddin et al., 2019).

In the current study, a subtractive genomics based metabolic pathways analysis was applied for the prediction of drug targets against the clinically relevant *S. pneumoniae* serotype 14. In the present study, metabolic pathway analysis of *S. pneumoniae* against human host was performed that resulted in the prediction of 25 metabolic pathways uniquely present in *S. pneumoniae*. The proteins involved in these pathways were retrieved and further studied for homologous proteins identification and also to identify proteins that are only found in pathogens. The computational subtractive genomics is a powerful tool to prioritize those proteins that are essential and play a role in the pathogenicity. However, the unique pathways found exclusively in bacteria may still share the common proteins that are found in the bacterial pathogens as well as in human host. Similarly, there are common pathways between the pathogen and human host that may also have unique proteins and those unique proteins may also act as drug targets. That common proteins may still be looked for and studied to find the drug targets since the mere similarity among proteins is not the only criterion to exclude them from the search of drug targets. There are many examples of successful drugs which target common proteins of the pathogens that are shared by the human host. One such drug target is a bacterial enzyme known as *Pantothenate synthetase* that has a homolog present in human host, however, with low similarity (∼40%) and it is safely considered as a drug target for numerous other bacterial pathogens including *M. tuberculosis* (Suresh et al., 2020; Rahman et al., 2021). It is the matter of focus of the current study that we considered the unique and non-homologous proteins only from the bacterial pathogen to proceed in our work pipeline. This could be considered as a limitation of the current applied protocol. However, one may explore the unique pathways for common proteins and also the common pathways for the unique proteins depending upon the similarity thresholds.

Furthermore, the filtration for essential proteins/genes were performed by the DEG database. The essential genes from the DEG database are identified by *in vitro* rich medium for the growth of the bacteria, therefore, it may result in identifying those proteins which may not be essential during the *in vivo* (i.e., in the host) infection phase. This is the major limitation of working with the DEG. Even with this limitation the DEG has been well studied in literature examples and produced the reliable results that led to the identification of novel *in vivo* drug targets (Umland et al., 2012).

In the next step, the drug target-like proteins were searched with the help of DrugBank database. Consequently, a total of 47 essential druggable and unique proteins were prioritized as potential drug targets against *S. pneumoniae* serotype 14. Finally, only two enzymes out of the 47 shortlisted proteins were selected for further ligand discovery as potential drug candidates against *S. pneumonia*. The selected two proteins are 4-oxalocrotonate tautomerase and sensor histidine kinases. These two proteins were selected because of their role in benzoate degradation and two-component system as both are critical for the growth and survival of the bacteria (Gupta et al., 2020). Though the focus of the current study is on the above mentioned two proteins, the other proteins can also be equally considered for further study to characterize them as potential drug targets against which lead compounds can be developed as potent drugs.

The 4-oxalocrotonate tautomerase enzyme is involved in benzonate and xylene degradation pathways which is unique to *S. pneumonia*. The benzonate and xylene degradation pathways degrade the aromatic compounds and amino acids into essential hydrocarbons to fulfill its carbon and energy requirements (Medellin et al., 2020; Baas et al., 2021). On the other hand, the sensor histidine kinase is involved in the Two-Component System (TCS) of *S. pneumonia* and regulates certain physiological conditions. The TCS is a basic signaling mechanism that allows bacteria to detect environmental signals and develop an appropriate stress response by expressing genes that allow for environmental adaptability. The function of these proteins elaborate their importance and hence can be considered as potential drug targets (Rosales-Hurtado et al., 2020). The shortlisted proteins were modeled through modeler and validated through PROCHECK, PSIPRED, and ProSA. Furthermore, the inhibitors, i.e., XAM (against CiaH) and DPM (for XylH), were predicted against through probis server and were screened using AutoDock tool. Subsequently, the interaction analysis was performed through MOE tool. These compounds showed favorable potency against each respective protein with the estimated binding affinities of –5.09 kcal/mol for XAM against CiaH and –7.59 kcal/mol of DPM against XylH. The results we achieved in the current study are presented here and are open for the experimental validation of compounds against respective drug targets in future by the scientific community.

The above study predicted the essential proteins that may most likely act as potential drug targets due to their involvement in essential pathways of *S. pneumoniae* (Lo et al., 2019). Additionally, the metabolic pathways associated with the cytoplasmic proteins may be used to formulate the drug targets, whereas the membrane-associated proteins may be used to formulate peptide vaccines (Masomian et al., 2020). All of the remaining non-homologous essential proteins, on the other hand, might be good therapeutic targets. The vaccines and therapies that target the activities of these proteins may eventually lead to the destruction and eradication of pathogen from the respective hosts. The current study covered all important and potent pharmacological targets in *S. pneumoniae* that will certainly aid future researchers in developing effective treatment or vaccine candidates. Therefore, various other computational approaches along with this approach in collaboration with experimental researchers could be used in the future to produce potential therapeutic strategy not only against *S. pneumoniae* but for other pathogens too.

## Conclusion

The analysis of the genome and proteome of many pathogens has aided the prediction of drug targets. In the current study, a subtractive genomic-based metabolic pathway analysis approach was applied to predict non-homologous essential druggable proteins against *S. pneumoniae* participating in the unique metabolic pathway. However, all the non-homologous essential proteins may also act as promising drug targets. Targeting these protein’s functions through novel drug candidates may lead to the destruction and the eradication of pathogen from the respective host. The analysis and results of the study covered all essential, potent drug targets in *S. pneumoniae* and thus it may facilitate future researchers to develop effective drug compounds and vaccines against strain-specific *S. pneumoniae* serotype 14.

## Data Availability Statement

The original contributions presented in the study are included in the article/Supplementary Material, further inquiries can be directed to the corresponding authors.

## Author Contributions

RU, AK, and AA-H conceived and designed the study. KK and KJ performed data collection and analysis and contributed to drafting the manuscript. RU provided technical and material support and supervised the study. All authors approved the final version of the manuscript.

## Conflict of Interest

The authors declare that the research was conducted in the absence of any commercial or financial relationships that could be construed as a potential conflict of interest.

## Publisher’s Note

All claims expressed in this article are solely those of the authors and do not necessarily represent those of their affiliated organizations, or those of the publisher, the editors and the reviewers. Any product that may be evaluated in this article, or claim that may be made by its manufacturer, is not guaranteed or endorsed by the publisher.

## Footnotes

1
https://proteins.plus/
