# Supplementary material for: Comparative Metabolic Pathways Analysis and Subtractive Genomics Profiling to Prioritize Potential Drug Targets Against Streptococcus pneumoniae
Source: Front Microbiol. 2022 Feb 10;12:796363. doi: 10.3389/fmicb.2021.796363 (PMC8866961; doi:10.3389/fmicb.2021.796363)

**Supplementary Table 1: Shortlisted 105 essential proteins against *S. pneumoniae***

| S. No | Protein IDs | Protein Names | DEG target |
| --- | --- | --- | --- |
| 1 | B2IM49 | Aspartate--ammonia ligase | **DEG10050178** |
| 2 | B2IQY9 | Chromosomal replication initiator protein DnaA | **DEG10210001, DEG10370001,**  **DEG10320309** |
| 3 | B2INJ3 | D-alanyl carrier protein | **DEG10170092** |
| 4 | B2IN76 | UDP-N-acetylmuramoylalanine--D-glutamate ligase | **DEG10070140,**  **DEG10210060,**  **DEG10380174,**  **DEG10360225** |
| 5 | B2IN77 | UDP-N-acetylglucosamine--N-acetylmuramyl-(pentapeptide)pyrophosphoryl-undecaprenol N-acetylglucosamine transferase | **DEG10070141,**  **DEG10210061,**  **DEG10370159,**  **DEG10420103** |
| 6 | B2ISX9 | Glutamate racemase | **DEG10070223,**  **DEG10210156,**  **DEG10370048,**  **DEG10420252** |
| 7 | B2ISB6 | Accessory Sec system protein translocase | **DEG10170314,**  **DEG10130412,**  **DEG10210025,**  **DEG10400558** |
| 8 | B2IM77 | Undecaprenyl-diphosphatase | **DEG10070029,**  **DEG10460395,**  **DEG10200444,**  **DEG10440120** |
| 9 | B2IQY8 | UDP-N-acetylmuramate--L-alanine ligase | **DEG10070186,**  **DEG10370046,**  **DEG10020208,**  **DEG10400435,**  **DEG10340060,**  **DEG10400378** |
| 10 | B2IQW9 | ATP synthase epsilon chain | **DEG10070181,**  **DEG10210081,**  **DEG10080205,**  **DEG10180547** |
| 11 | B2IQX6 | ATP synthase subunit c | **DEG10210074,**  **DEG10420150** |
| 12 | B2IN15 | 2,3,4,5-tetrahydropyridine-2,6-dicarboxylate | **DEG10010093,**  **DEG10130316,**  **DEG10410139** |
| 13 | B2IQK6 | UDP-N-acetylenolpyruvoylglucosamine reductase | **DEG10070176,**  **DEG10380132,**  **DEG10450235** |
| 14 | B2IR75 | 4-hydroxy-tetrahydrodipicolinate reductase | **DEG10010156,**  **DEG10360274,**  **DEG10080085,**  **DEG10220433** |
| 15 | B2IRS6 | D-alanine--D-alanine ligase | **DEG10070207,**  **DEG10020230,**  **DEG10280452,**  **DEG10220150,**  **DEG10080120** |
| 16 | B2IRV7 | Protein translocase subunit SecA | **DEG10210052,**  **DEG10270575,**  **DEG10250368** |
| 17 | B2ILR1 | Penicillin-binding protein 1A | **DEG10380182,**  **DEG10170188** |
| 18 | B2IMX4 | Histidine kinase | **DEG10470503,**  **DEG10470177** |
| 19 | B2ILQ9 | Oligopeptide ABC transporter, | **DEG10470296,**  **DEG10450345** |
| 20 | B2INE1 | PTS system, IIABC components | **DEG10020290** |
| 21 | B2IRQ1 | PTS system IIABC components | **DEG10320195,**  **DEG10060051** |
| 22 | B2ISZ0 | Oligopeptide ABC transporter, | **DEG10470297,**  **DEG10080052,**  **DEG10450345** |
| 23 | B2ISQ4 | Penicillin-binding protein 2X | **DEG10210169,**  **DEG10010101,**  **DEG10240391,**  **DEG10290370** |
| 24 | B2IRG2 | DNA-binding response regulator | **DEG10340401,**  **DEG10260070,**  **DEG10300014,**  **DEG10030206** |
| 25 | B2IRE6 | PTS system, IIA component | **DEG10280041,**  **DEG10410094** |
| 26 | B2IRS8 | Penicillin-binding protein 2B | **DEG10470219,**  **DEG10330012** |
| 27 | B2ISR3 | PTS system, IIB component | **DEG10470465,**  **DEG10030262,**  **DEG10180293** |
| 28 | B2IND2 | Branched-chain amino acid ABC transporter, | **DEG10350191,**  **DEG10470170,**  **DEG10350330** |
| 29 | B2IML0 | PTS system, IIA component | **DEG10180603,**  **DEG10020243** |
| 30 | B2IR50 | UDP-N-acetylmuramoyl-L-alanyl-D-glutamate--L-lysine ligase | **DEG10460380,**  **DEG10410444,**  **DEG10190009** |
| 31 | B2IPT1 | PTS system IIA component, putative | **DEG10070172** |
| 32 | B2IQ07 | Phosphate acetyltransferase | **DEG10230230,**  **DEG10450182,**  **DEG10220243,**  **DEG10170051** |
| 33 | B2IPQ6 | Sensory box sensor histidine kinase | **DEG10170010,**  **DEG10380037,**  **DEG10130463** |
| 34 | B2IN03 | Phosphate ABC transporter, phosphate-binding | **DEG10300108,**  **DEG10420175,**  **DEG10210101** |
| 35 | B2IPU1 | PTS system, lactose-specific IIBC components | **DEG10030262** |
| 36 | B2IPR3 | Uncharacterized protein | **DEG10370128,**  **DEG10470063** |
| 37 | B2ISY9 | Oligopeptide ABC transporter, permease | **DEG10060059,**  **DEG10140104,**  **DEG10470017** |
| 38 | B2ISY8 | Oligopeptide ABC transporter, permease | **DEG10180213,**  **DEG10080053,**  **DEG10410149** |
| 39 | B2ISN1 | PTS system, IIA component | **DEG10180292** |
| 40 | B2ISB2 | Protein translocase subunit SecA | **DEG10020297,**  **DEG10370198**  **DEG10370198**  **DEG10150247**  **DEG10190021** |
| 41 | B2IRA4 | Mur ligase family protein | **DEG10210083,**  **DEG10370119**  **DEG10170272**  **DEG10290363** |
| 42 | B2IMT2 | Beta-lactam resistance factor | **DEG10370072**  **DEG10170181**  **DEG10370073**  **DEG10420111** |
| 43 | B2IP77 | Uncharacterized protein | **DEG10500049,**  **DEG10220144** |
| 44 | B2IMJ7 | PTS system, IIB component | **DEG10030262**  **DEG10470465**  **DEG10180293** |
| 45 | B2IPH4 | 4-oxalocrotonate tautomerase | **DEG10170180** |
| 46 | B2IN55 | Ascorbate-specific PTS system enzyme IIC | **DEG10110054** |
| 47 | B2IRV3 | Alanine racemase | **DEG10210054**  **DEG10010072**  **DEG10270600** |
| 48 | B2IMK2 | Preprotein translocase subunit YajC | **DEG10240151**  **DEG10390177**  **DEG10120247** |
| 49 | B2IMJ8 | PTS system, IIA component | **DEG10180292** |
| 50 | B2IN01 | Response regulator | **DEG10010263**  **DEG10070064**  **DEG10210141**  **DEG10430427**  **DEG10380148**  **DEG10230052** |
| 51 | B2ILS7 | Sensor histidine kinase | **DEG10020213**  **DEG10470265**  **DEG10270563**  **DEG10100506** |
| 52 | B2IRU0 | PTS system, IIBC components | **DEG10020290**  **DEG10140139 DEG10140140** |
| 53 | B2IPU0 | PTS system, lactose-specific IIA component | **DEG10180292** |
| 54 | B2ISS6 | Anthranilate synthase component 1 | **DEG10250328**  **DEG10280391**  **DEG10250185**  **DEG10250633** |
| 55 | B2IRV6 | Phospho-2-dehydro-3-deoxyheptonate aldolase | **DEG10290252**  **DEG10180285**  **DEG10400648** |
| 56 | B2ISP7 | Preprotein translocase, YajC subunit | **DEG10240151**  **DEG10220304**  **DEG10200293** |
| 57 | B2IMI4 | Penicillin-binding protein 2A | **DEG10170188**  **DEG10310062**  **DEG10410234**  **DEG10050303** |
| 58 | B2ISH4 | PTS system, IIA component | **DEG10180292** |
| 59 | B2IQH8 | Homoserine dehydrogenase | **DEG10400530**  **DEG10250232**  **DEG10030465** |
| 60 | B2IMP9 | PTS system, beta-glucosides-specific IIABC | **DEG10320195**  **DEG10020290** |
| 61 | B2IPC2 | Diacylglycerol kinase | **DEG10380058**  **DEG10080114** |
| 62 | B2IMH5 | Sensor histidine kinase, putative | **DEG10100506**  **DEG10270563**  **DEG10470265** |
| 63 | B2IPM7 | Phosphoenolpyruvate-protein | **DEG10020092**  **DEG10430260**  **DEG10200181** |
| 64 | B2IRA5 | GATase cobBQ-type domain-containing protein | **DEG10380125**  **DEG10250723**  **DEG10170271** |
| 65 | B2IML8 | Acetate kinase | **DEG10020202**  **DEG10180359**  **DEG10060294** |
| 66 | B2IS14 | Riboflavin biosynthesis protein RibD | **DEG10290303**  **DEG10230238**  **DEG10190147** |
| 67 | B2ISY2 | Trehalose PTS system, IIABC components | **DEG10330065**  **DEG10020290**  **DEG10320195** |
| 68 | B2IND4 | Branched-chain amino acid ABC transporter, permease protein | **DEG10350331** |
| 69 | B2IPQ5 | DNA-binding response regulator | **DEG10210141**  **DEG10270162** |
| 70 | B2IM45 | UDP-N-acetylglucosamine | **DEG10330235**  **DEG10430112**  **DEG10280119** |
| 71 | B2IN17 | Penicillin-binding protein 1B | **DEG10250014**  **DEG10410256**  **DEG10440098** |
| 72 | B2INS3 | Sensor histidine kinase CiaH | **DEG10270175**  **DEG10180268**  **DEG10440058** |
| 73 | B2ISM9 | PTS system, IIB component | **DEG10030262**  DEG10470465 |
| 74 | B2IQ25 | UDP-N-acetylglucosamine | **DEG10030507** |
| 75 | B2IR47 | Oligopeptide ABC transporter, | **DEG10470297** |
| 76 | B2ILU2 | PTS system, mannitol-specific IIA component | **DEG10470314** |
| 77 | B2IML2 | Ascorbate-specific PTS system enzyme IIC | **DEG10110054** |
| 78 | B2IML5 | Membrane protein insertase YidC | **DEG10080290**  **DEG10230024**  **DEG10030001** |
| 79 | B2ISR2 | Tyrosine-protein phosphatase | **DEG10070015** |
| 80 | B2ILY7 | Acetolactate synthase small subunit | **DEG10100484**  **DEG10280098** |
| 81 | B2INS2 | DNA-binding response regulator CiaR | **DEG10410226** |
| 82 | B2ILU0 | PTS system, mannitol-specific IIBC components | **DEG10070132** |
| 83 | B2ISP2 | PTS system, IIA component | **DEG10410094**  **DEG10280041** |
| 84 | B2ISL0 | PTS system, mannose-specific IIAB components | **DEG10180464**  **DEG10280041** |
| 85 | B2IS60 | Protein translocase subunit SecY | **DEG10470013**  **DEG10390236**  **DEG10340461** |
| 86 | B2ILV9 | Aspartokinase OS=Streptococcus pneumoniae | **DEG10500188**  **DEG10270648**  **DEG10290290** |
| 87 | B2IMS2 | Fructose-bisphosphate aldolase | **DEG10380216**  **DEG10140030** |
| 88 | B2IRV5 | Phospho-2-dehydro-3-deoxyheptonate aldolase | **DEG10180285** |
| 89 | B2IPC7 | Protein-export membrane protein SecG | **DEG10210143** |
| 90 | B2INJ4 | Protein DltB | **DEG10170091** |
| 91 | B2ISP4 | PTS system, IIB component | **DEG10180464** |
| 92 | B2IRS5 | UDP-N-acetylmuramoyl-tripeptide--D-alanyl-D-alanine ligase | **DEG10370152**  **DEG10170291** |
| 93 | B2IMI2 | Translocase | **DEG10210208**  **DEG10420297** |
| 94 | B2INJ2 | Protein DltD | **DEG10170093** |
| 95 | B2IMT1 | Beta-lactam resistance factor | **DEG10170335**  **DEG10380071**  **DEG10020285** |
| 96 | B2IRE3 | PTS system, IIB component | **DEG10180464** |
| 97 | B2IQ40 | Phosphoenolpyruvate carboxylase | **DEG10400580**  **DEG10440155** |
| 98 | B2IQL7 | Phosphate ABC transporter, phosphate-binding | **DEG10210101**  **DEG10110203**  **DEG10300108** |
| 99 | B2ISH5 | PTS system IIA component, putative | **DEG10470465** |
| 100 | B2ILS8 | DNA-binding response regulator | **DEG10380177**  **DEG10400471**  **DEG10250641** |
| 101 | B2ISF5 | Phosphotransferase system (PTS) | **DEG10180292** |
| 102 | B2INZ2 | D-alanyl-D-alanine carboxypeptidase | **DEG10410383**  **DEG10440011** |
| 103 | B2IPH1 | Aspartate-semialdehyde dehydrogenase | **DEG10500286**  **DEG10240377**  **DEG10150120** |
| 104 | B2IP07 | PTS system, fructose specific IIABC | **DEG10020295**  **DEG10180477**  **DEG10020243** |
| 105 | B2IMV6 | PTS system IIA component, putative | **DEG10070172** |

**Supplementary Table 2: Shortlisted 47 drug target like proteins against *S. pneumoniae***

| S. No | Protein IDs | Protein Names | Drug Bank target |
| --- | --- | --- | --- |
| 1 | B2IN76 | UDP-N-acetylmuramoylalanine--D-glutamate ligase | DB01673, DB02314, DB03801, DB08105, DB08106, DB08107, DB08108 |
| 2 | B2IN77 | UDP-N-acetylglucosamine--N-acetylmuramyl-(pentapeptide) pyrophosphoryl-undecaprenol N-acetylglucosamine transferase | DB02196 |
| 3 | B2ISX9 | Glutamate racemase | DB08698, DB02174, DB02343, DB07937, DB08272 |
| 4 | B2IQY8 | UDP-N-acetylmuramate--L-alanine ligase | DB01673; DB03909; DB04395 |
| 5 | B2IQX6 | ATP synthase subunit c | DB04464 |
| 6 | B2IN15 | 2,3,4,5-tetrahydropyridine-2,6-dicarboxylate N-acetyltransferase | DB01856; DB01992; DB03134; DB03699; DB03905 |
| 7 | B2IQK6 | UDP-N-acetylenolpyruvoylglucosamine reductase | DB03147 |
| 8 | B2IR75 | 4-hydroxy-tetrahydrodipicolinate reductase | DB04267 |
| 9 | B2IRS6 | D-alanine--D-alanine ligase | DB00128; DB03459 |
| 10 | B2ILR1 | Penicillin-binding protein 1A | DB01150; DB05659 |
| 11 | B2IMX4 | Histidine kinase | DB04395 |
| 12 | B2ILQ9 | Oligopeptide ABC transporter | B07365 |
| 13 | B2ISZ0 | Oligopeptide ABC transporter | DB07365 |
| 14 | B2ISQ4 | Penicillin-binding protein 2X | DB03190, DB00319, DB01147 |
| 15 | B2IRG2 | DNA-binding response regulator | DB02355; DB02596; DB07706 |
| 16 | B2IRS8 | Penicillin-binding protein 2B | DB00319; DB00415; DB00456; DB00485; DB00493; DB00567; DB00607; DB00713; DB00739; DB01066; DB01140; DB01163; DB01212; DB01331; DB01603; DB03313; DB08795 |
| 17 | B2ISR3 | PTS system, IIB component | B03544 |
| 18 | B2IND2 | Branched-chain amino acid ABC transporter | B02121; DB02736 |
| 19 | B2IR50 | UDP-N-acetylmuramoyl-L-alanyl-D-glutamate--L-lysine ligase | B02314; DB03590; DB03801 |
| 20 | B2IQ07 | Phosphate acetyltransferase | DB02897 |
| 21 | B2IPQ6 | Sensory box sensor histidine kinase | DB02355; DB02596; DB07706 |
| 22 | B2IRA4 | Mur ligase family protein | DB01673; DB03909; DB04395 |
| 23 | B2IMJ7 | PTS system, IIB component | DB03544 |
| 24 | B2IPH4 | 4-oxalocrotonate tautomerase | DB02005 |
| 25 | B2IRV3 | Alanine racemase | DB03327; DB03579; DB03766; DB03801; DB04467 |
| 26 | B2IN01 | Response regulator | DB01972 |
| 27 | B2ISS6 | Anthranilate synthase | DB01942 |
| 28 | B2IRV6 | Phospho-2-dehydro-3-deoxyheptonate aldolase | DB01819; DB02726 |
| 29 | B2IMI4 | Penicillin-binding protein 2A | DB00415; DB00456; DB00485; DB00493; DB00567; DB00607; DB00713 |
| 30 | B2IPM7 | Phosphoenolpyruvate-protein phosphotransferase | DB08357 |
| 31 | B2IML8 | Acetate kinase | DB01942; DB03909 |
| 32 | B2IPQ5 | DNA-binding response regulator | DB02355; DB02596; DB07706 |
| 33 | B2IM45 | UDP-N-acetylglucosamine 1-carboxyvinyltransferase | DB01879; DB02435; DB02995; DB03089; DB04174; DB04474 |
| 34 | B2IN17 | Penicillin-binding protein 1B | DB00415; DB00456; DB00485; DB00493; DB00567; DB00607; DB00713 |
| 35 | B2INS3 | Sensor histidine kinase CiaH | DB02355; DB02596; DB07706 |
| 36 | B2IQ25 | UDP-N-acetylglucosamine 1-carboxyvinyltransferase | DB03397 |
| 37 | B2IR47 | Oligopeptide ABC transporter, oligopeptide-binding protein AliB | DB07365 |
| 38 | B2INS2 | DNA-binding response regulator CiaR | DB02355; DB02596 |
| 39 | B2IMS2 | Fructose-bisphosphate aldolase | DB03026 |
| 40 | B2IRV5 | Phospho-2-dehydro-3-deoxyheptonate aldolase | DB01819; DB02726 |
| 41 | B2IRS5 | UDP-N-acetylmuramoyl-tripeptide--D-alanyl-D-alanine ligase | DB06970 |
| 42 | B2IQ40 | Phosphoenolpyruvate carboxylase | DB04317 |
| 43 | B2IQL7 | Phosphate ABC transporter, phosphate-binding protein, putative | DB02799;  DB02831 |
| 44 | B2ISH5 | PTS system, IIB component | DB03544 |
| 45 | B2ILS8 | DNA-binding response regulator | DB03793 |
| 46 | B2INZ2 | D-alanyl-D-alanine carboxypeptidase | DB00415; DB00438; DB00447; DB00456; DB00485; DB00567; DB00607 |
| 47 | B2IPH1 | Aspartate-semialdehyde dehydrogenase | DB03461;  DB03502; DB04498 |

**Supplementary Figure 1.** Multiple Sequence Alignment Analysis of XylH proteins with similar proteins from other serotypes of *S. pneumoniae*, (B) And their related percent identity matrix.


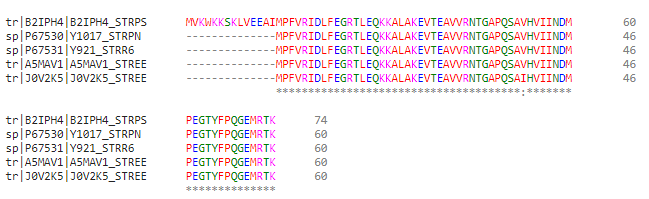


**A**

| Proteins | B2IPH4 | P67530 | P67531 | A5MAV1 | J0V2K5 |
| --- | --- | --- | --- | --- | --- |
| B2IPH4  **B** | 100.00 | 100.00 | 100.00 | 100.00 | 98.33 |
| P67530 | 100.00 | 100.00 | 100.00 | 100.00 | 98.33 |
| P67531 | 100.00 | 100.00 | 100.00 | 100.00 | 98.33 |
| A5MAV1 | 100.00 | 100.00 | 100.00 | 100.00 | 98.33 |
| J0V2K5 | 98.33 | 98.33 | 98.33 | 98.33 | 100.00 |

**Supplementary Figure 2.** Multiple Sequence Alignment Analysis of CiaH proteins with similar proteins from other serotypes of *S. pneumoniae*, (B) And their related percent identity matrix.

**A**


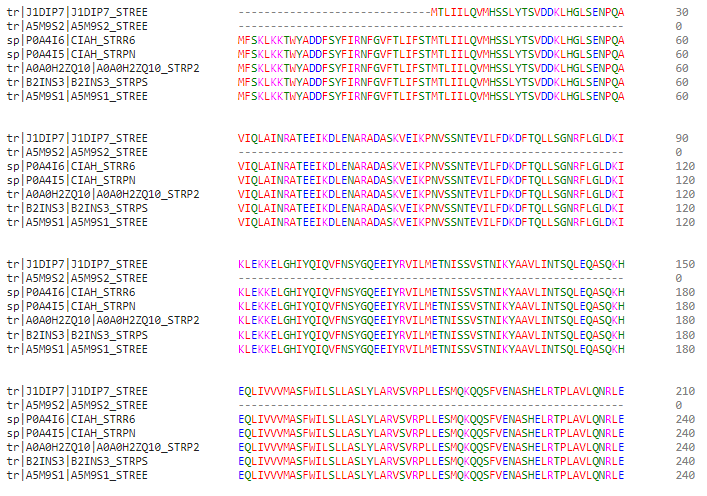

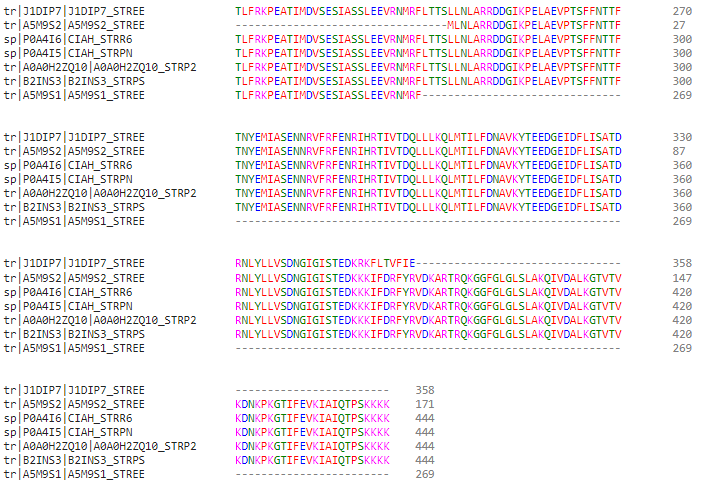


**B**

| Proteins | J1DIP7 | A5M9S2 | P0A4I6 | P0A4I5 | A0A0H2ZQ10 | B2INS3 | A5M9S1 |
| --- | --- | --- | --- | --- | --- | --- | --- |
| J1DIP7 | 100.00 | 93.04 | 98.04 | 98.04 | 98.04 | 98.04 | 100.00 |
| A5M9S2 | 93.04 | 100.00 | 99.42 | 99.42 | 99.42 | 99.42 | -nan |
| P0A4I6 | 98.04 | 99.42 | 100.00 | 100.00 | 100.00 | 100.00 | 100.00 |
| P0A4I5 | 98.04 | 99.42 | 100.00 | 100.00 | 100.00 | 100.00 | 100.00 |
| A0A0H2ZQ10 | 98.04 | 99.42 | 100.00 | 100.00 | 100.00 | 100.00 | 100.00 |
| B2INS3 | 98.04 | 99.42 | 100.00 | 100.00 | 100.00 | 100.00 | 100.00 |
| A5M9S1 | 100.00 | -nan | 100.00 | 100.00 | 100.00 | 100.00 | 100.00 |

**Supplementary Figure 3.** Secondary structure validation through PSIPRED predicts the positions for helixes and beta sheets for (A) XylH (B) and CiaH.

**A**


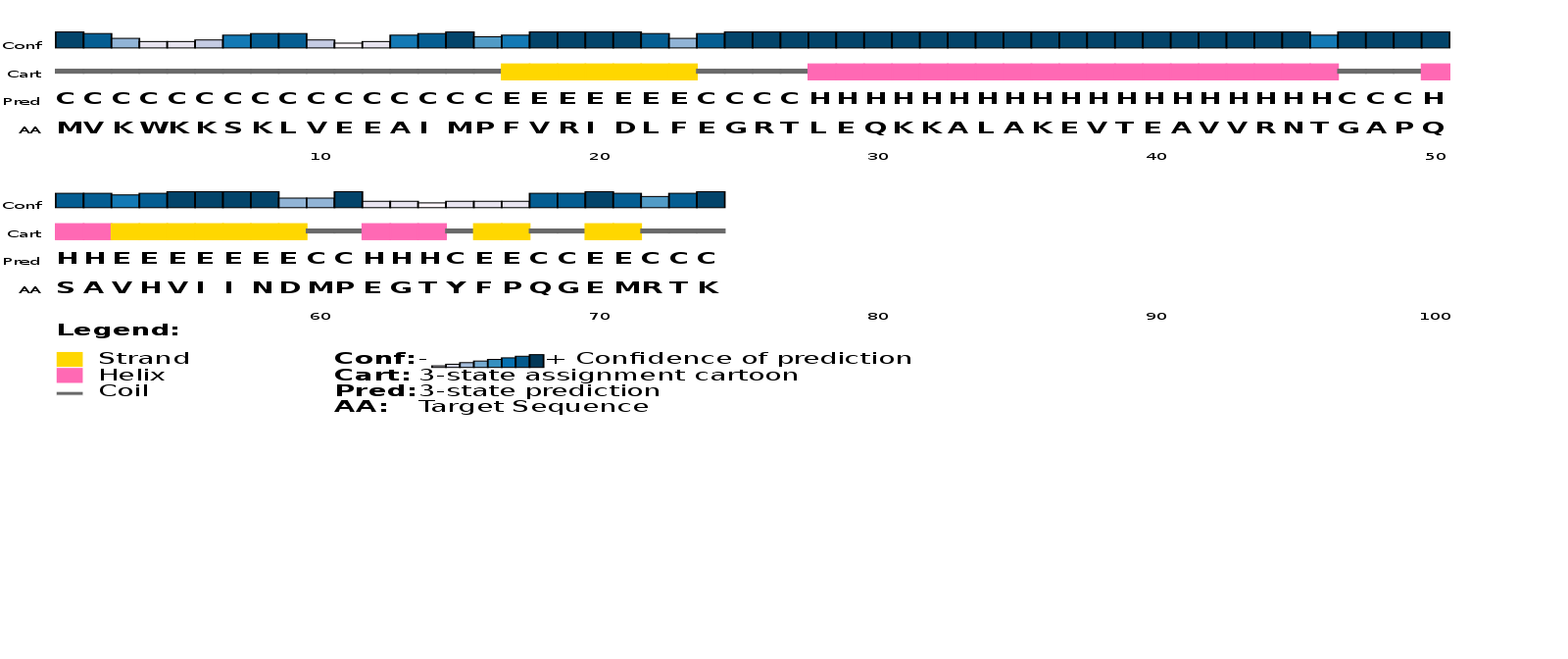


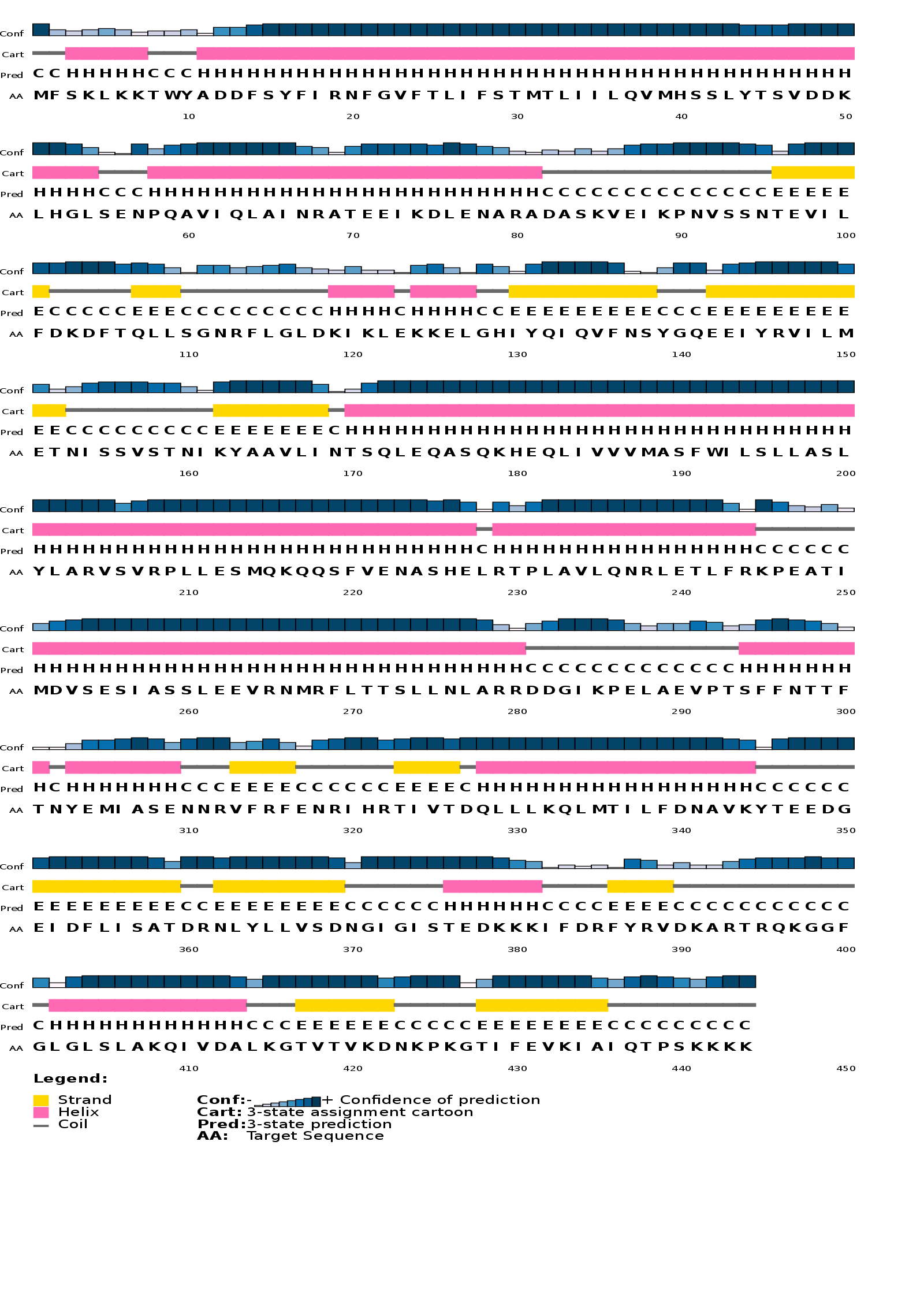


**B**

**Supplementary Figure 4.** Ramachandran Plot generated through PROCHECK shows 84% residues in the favored region for XylH protein (A) and 93% residues in the favorable region for CiaH (B).

**A**


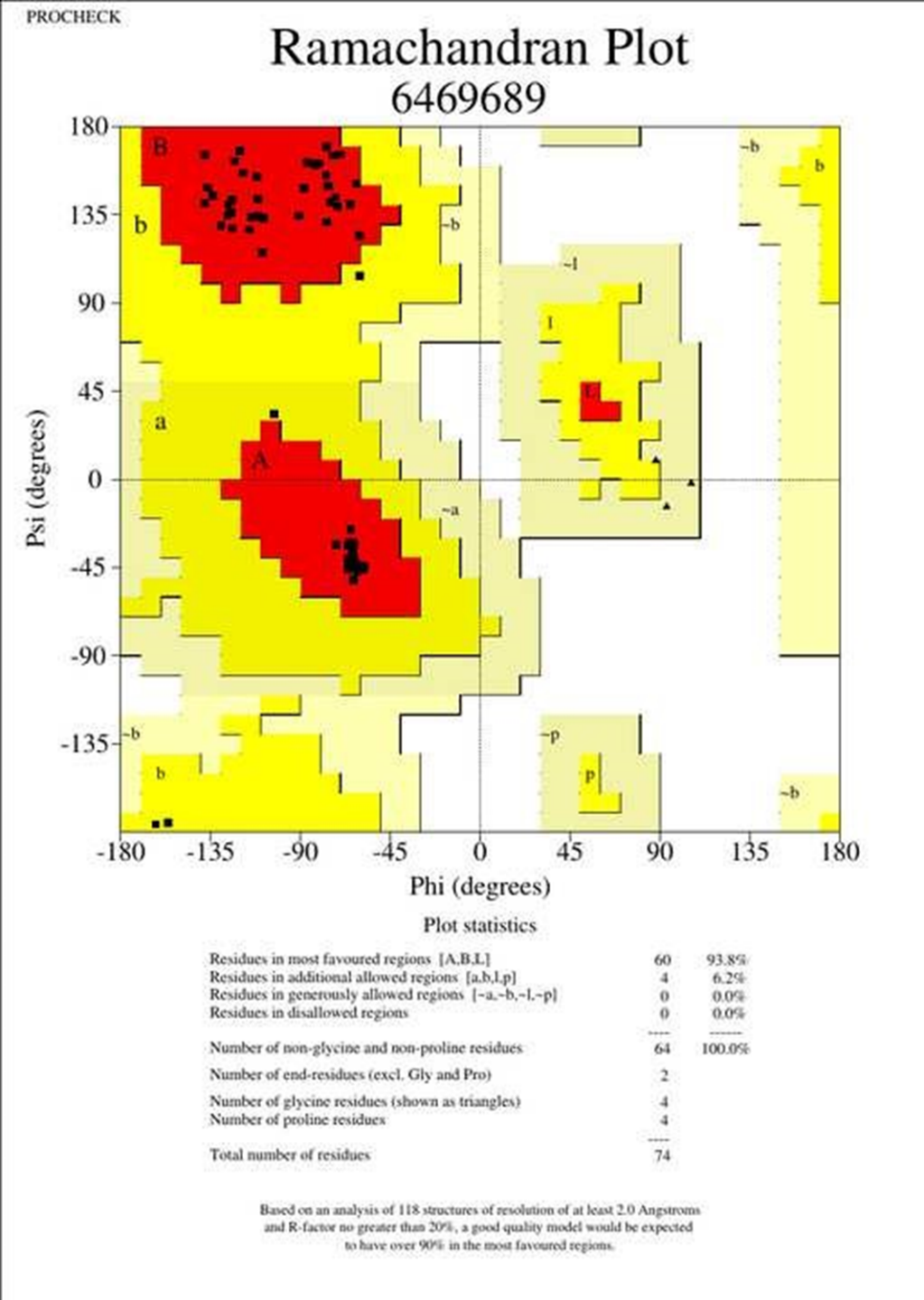


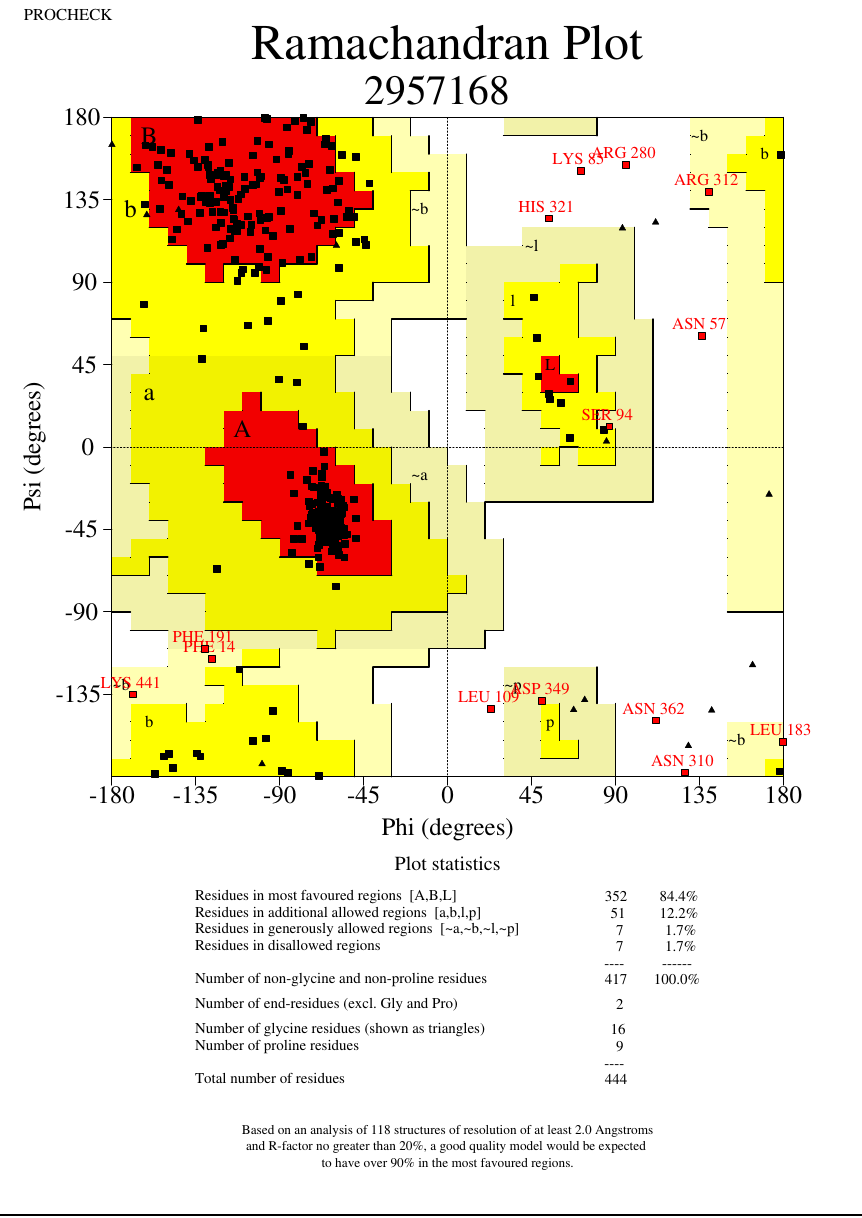


**B**

**Supplementary Figure 5**. Z-score plot generated through ProSA web for (A) XylH protein and (B) CiaH modeled structure showing a z-score value of -2.66 and -4.62 respectively.

**A**


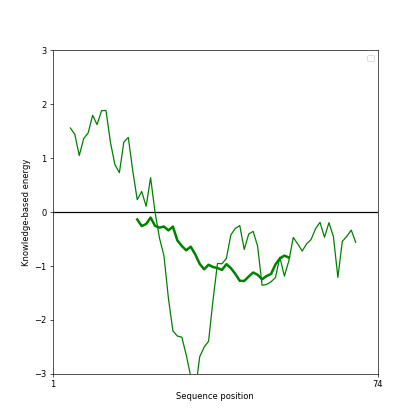

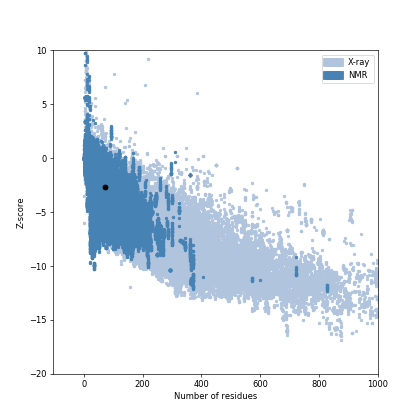

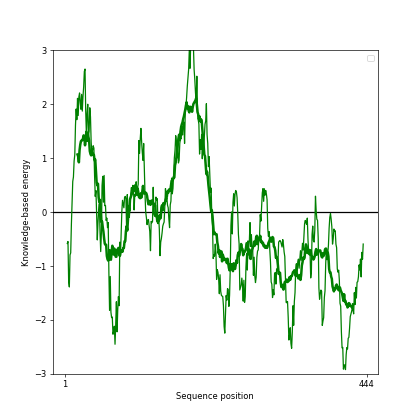

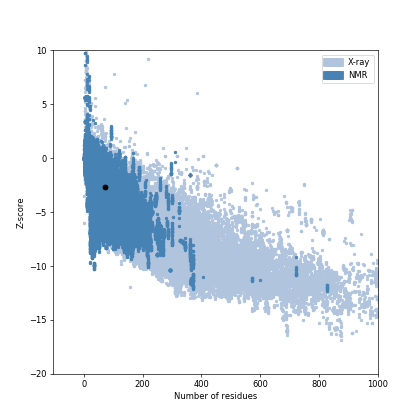


**B**

**Supplementary Figure 6. DogSite Scorer:** Active site prediction for XylH protein through Dogsite Scorer (A) shows predicted binding site whereas (B) for CiaH protein through Dogsite Scorer.

**
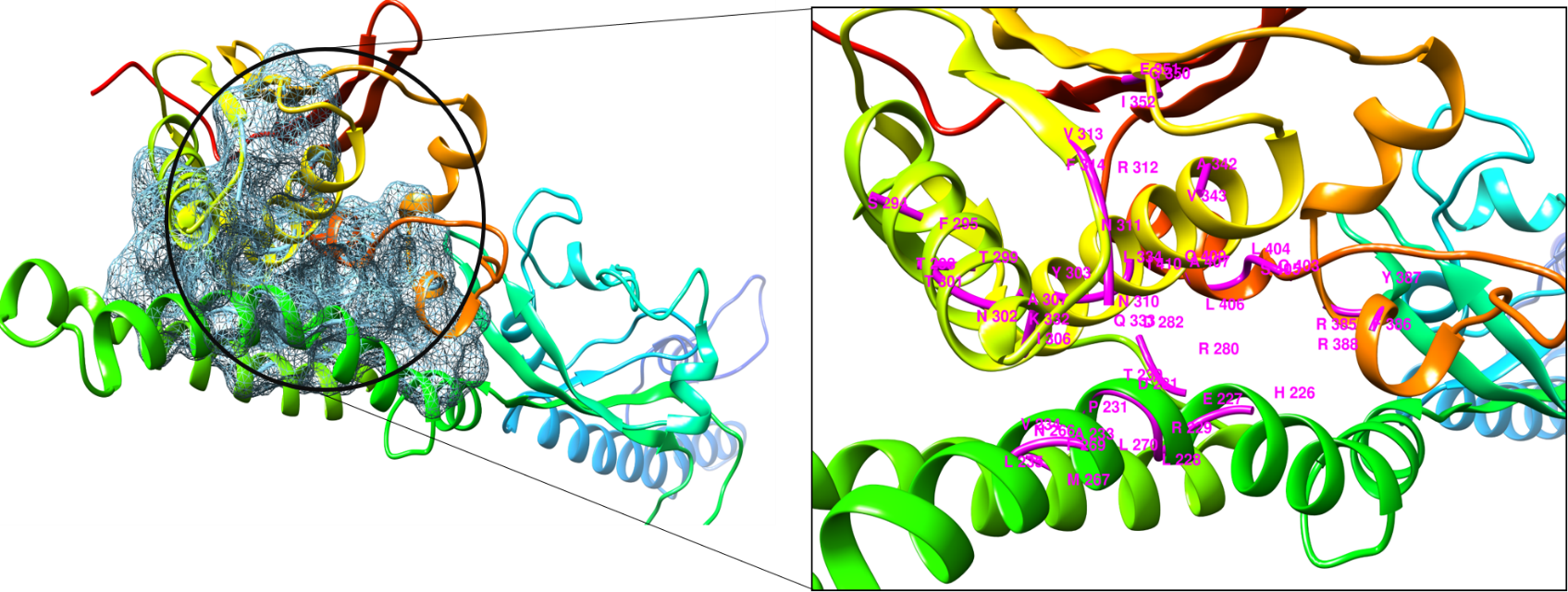
**
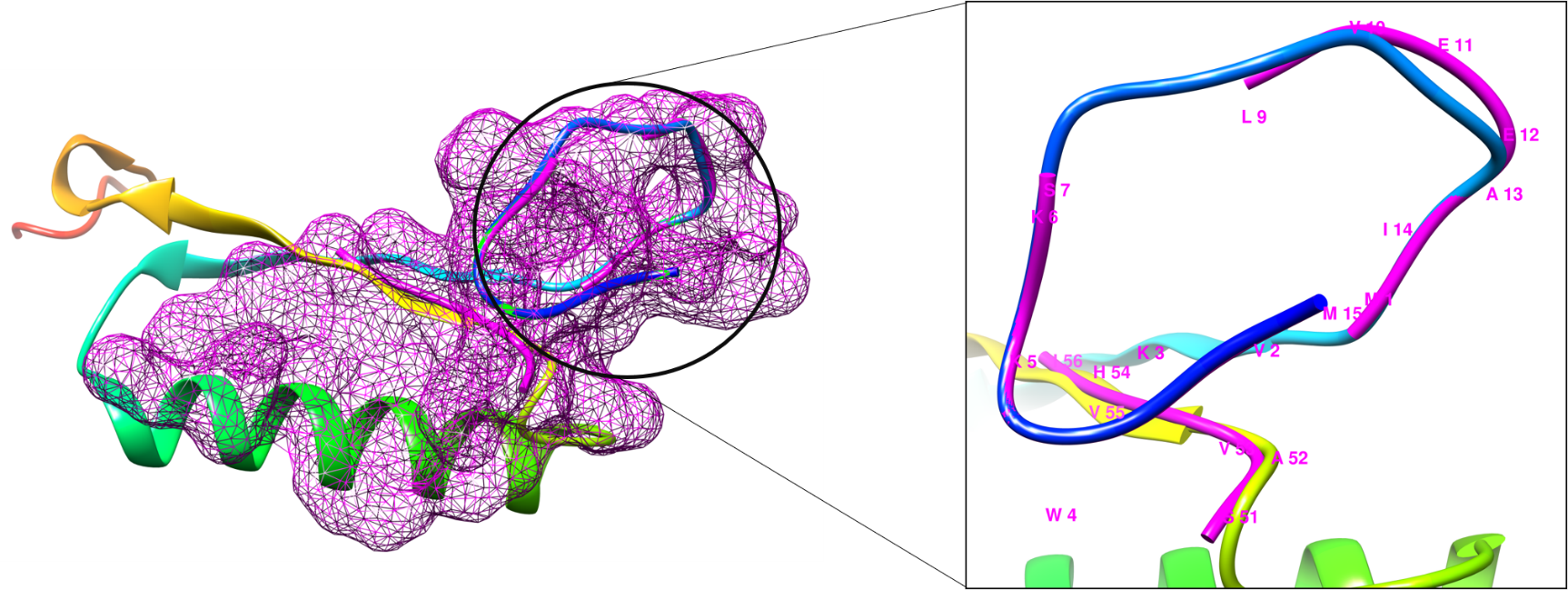


**B**

**A**

**Supplementary Figure 7. Identified Ligands:** ligands identified for drug targets through Probis server (A) DIPYRROMETHANE COFACTOR (DPM) IUPAC names 3-[5-{[3-(2-carboxyethyl)-4-(carboxymethyl)-5-methyl-1H-pyrrol-2-yl]methyl}-4-(carboxymethyl)-1H-pyrrol-3-yl]propanoic acid, ligand identified agaisnt XylH protein whereas (B) Amycolamicin (XAM), IUPAC names as, (1R,4aS,5S,6S,8aR)-5-{[(5S)-1-(3-O-acetyl-4-O-carbamoyl-6-deoxy-2-O-methyl-alpha-L-talopyranosyl)-4-hydroxy-2-oxo-5-(propan-2-yl)-2,5-dihydro-1H-pyrrol-3-yl]carbonyl}-6-methyl-4-methylidene-1,2,3,4,4a,5,6,8a-octahydronaphthalen-1-yl-2,6-dideoxy-3-C-[(1S)-1-{[(3,4-dichloro-5-methyl-1H-pyrrol-2-yl)carbonyl]amino}ethyl]-beta-D-ribo-hexopyranoside


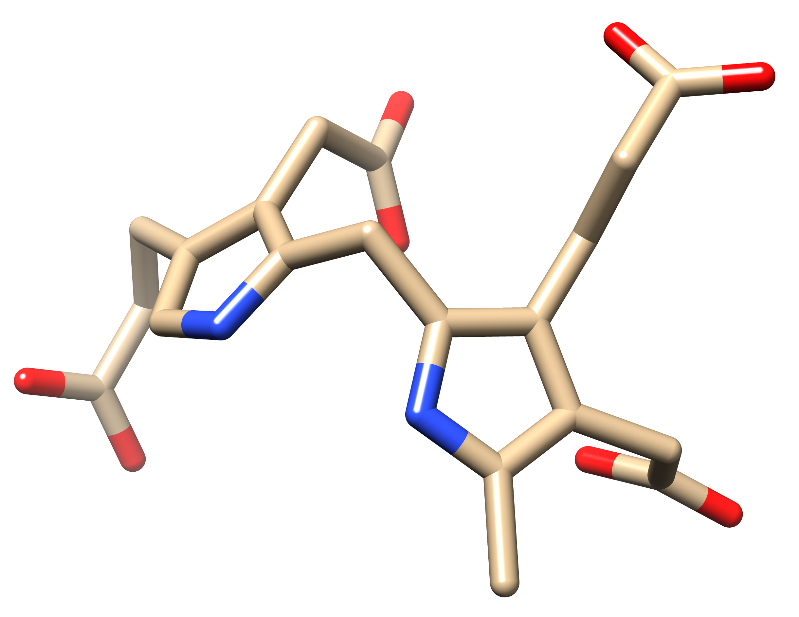


**A**

**B**


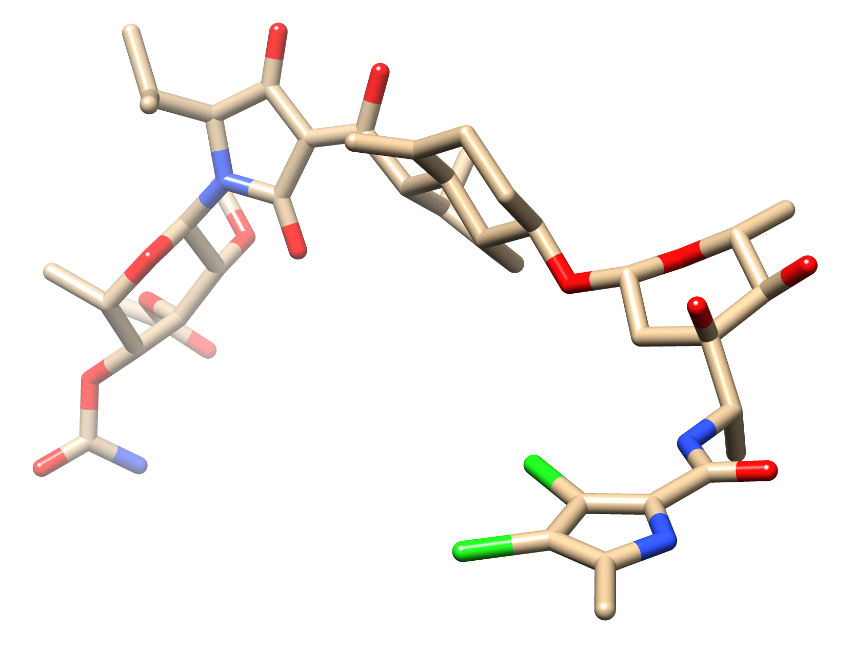

Supplement: Supplementary file 1 [file Data_Sheet_1.docx]
