# Supplementary material for: Comparative Metabolic Pathways Analysis and Subtractive Genomics Profiling to Prioritize Potential Drug Targets Against Streptococcus pneumoniae
Source: Front Microbiol. 2022 Feb 10;12:796363. doi: 10.3389/fmicb.2021.796363 (PMC8866961; doi:10.3389/fmicb.2021.796363)
Supplement: Supplementary file 2 [file Data_Sheet_2.docx]

UniProt Human accession number: [UP000005640](https://www.uniprot.org/proteomes/UP000005640)

UniProt *Streptococcus* *pneumoniae* accession number: [UP000001682](https://www.uniprot.org/proteomes/UP000001682) (strain CGSP14)
