# Supplementary figures and images for: Comparative Metabolic Pathways Analysis and Subtractive Genomics Profiling to Prioritize Potential Drug Targets Against Streptococcus pneumoniae
Source: Front Microbiol. 2022 Feb 10;12:796363. doi: 10.3389/fmicb.2021.796363 (PMC8866961; doi:10.3389/fmicb.2021.796363)

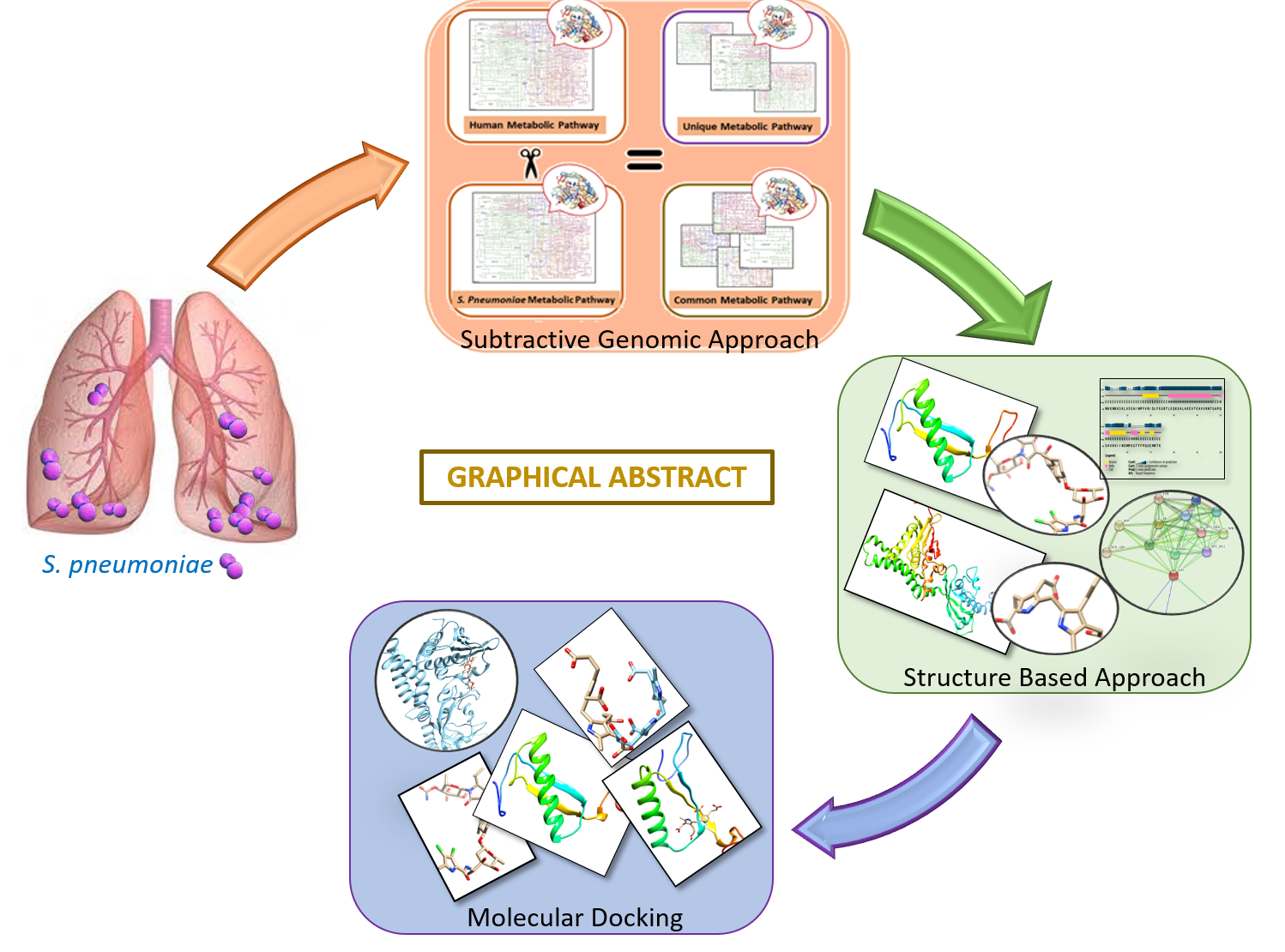

Supplement: Supplementary file 3 [file Image_1.PNG]
